# Heterogeneous Photoredox Catalysis Based on Silica Mesoporous Material and Eosin Y: Impact of Material Support on Selectivity of Radical Cyclization

**DOI:** 10.3390/molecules28020549

**Published:** 2023-01-05

**Authors:** Nadine Mahmoud, Jazia Awassa, Joumana Toufaily, Bénédicte Lebeau, T. Jean Daou, Morgan Cormier, Jean-Philippe Goddard

**Affiliations:** 1 Université de Haute-Alsace (UHA), Université de Strasbourg, CNRS, Laboratoire d’Innovation Moléculaire et Applications (LIMA) UMR 7042, 68100 Mulhouse, France; 2 Université de Haute Alsace, CNRS, IS2M, UMR 7361, 68100 Mulhouse, France; 3 Université de Strasbourg, F-67000 Strasbourg, France; 4 Laboratory of Materials, Catalysis, Environment and Analytical Methods, Faculty of Sciences I, Lebanese University, Campus Rafic Hariri, Beyrouth, Lebanon

**Keywords:** heterogeneous photoredox catalysis, mesoporous silica material, aza-Henry reaction, [2+2] cycloaddition, hybrid organic-inorganic material

## Abstract

Heterogenization of the photocatalyst appears to be a valuable solution to reach sustainable processes. Rapid and efficient synthesis of supported photocatalyst is still a remaining challenge and the choice of the support material is crucial. The present study aims at preparing a new generation of hybrid inorganic/organic photocatalysts based on silica mesoporous material and Eosin Y. These results highlight the influence of non-covalent interactions between the material support and the reagent impacting the selectivity of the reaction.

## 1. Introduction

**Scheme 1:**
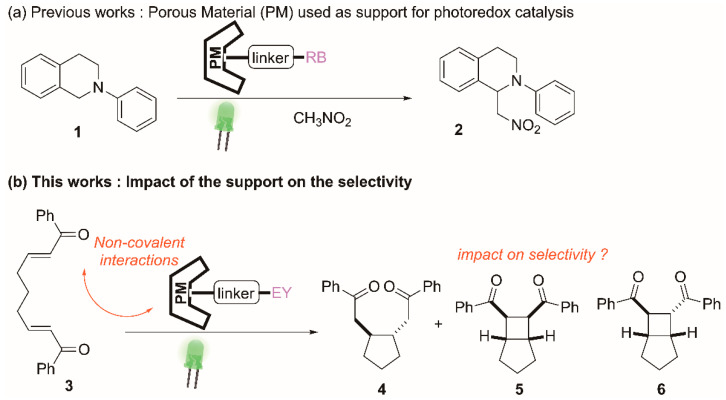
Organic/Inorganic heterogeneous silica-based photoredox catalyst for selective organic transformations.

Radical chemistry has always occupied a unique place in organic synthesis due to the extraordinary variety of possibilities it offers. Indeed, the manipulation of transitory and highly reactive radical intermediates offers efficient methods in oxidation and reduction reactions, C-C bond formation, introduction of heteroatoms and many other reactions. To address the challenging generation and control of radical species, photoredox catalysis has proved to be effective with the assistance of a photosensitizer and light (particularly visible light) [1,2,3,4,5,6,7,8,9,10,11,12,13,14,15,16,17]. This concept presents reduced environmental impact of chemical processes including: (i) reducing energy consumption by lowering temperature and pressure, and the use of sustainable sources of energy such as sunlight, and (ii) reducing waste production by limiting the use of organic solvents and improving selectivity. Conversion of low energy visible light into chemical energy via photoredox catalysis is an effective way to lower environmental impact. Recycling chemicals is crucial to reduce waste and cost especially for technology transfer to industry. A simple way to address this problem is to design heterogeneous catalytic systems that can be easily recycled from the reaction media [18,19]. The heterogeneous photoredox catalysis is now well established, a wide variety of heterogeneous photocatalyst is available [20,21]. Usually, an heterogeneous photocatalyst is composed of a dye (organometallic or organic) linked to an insoluble support (e.g., polymer, MOF, silica-based material) [22,23,24,25,26,27,28,29,30,31,32,33]. Recently we have contributed to this field by incorporating covalently Rose Bengal (RB) into silica mesoporous materials to reach hybrid inorganic/organic photocatalysts efficient to promote aza-Henry reactions (Scheme 1a) [34]. Up to now, the support was only designed to bring the heterogenization. Moreover, this heterogeneous platform should not only be considered as a heterogeneous anchor, but it should play an active role in modulating reactivity and selectivity according to the morphology, size, and nature of the material (Scheme 1b).

In this report, we present new hybrid photoredox catalysts set based on the conjugation of various porous and a non-porous (Stöber spheres) silica-based materials with Eosin Y dye. Both are linked by a covalent bond to afford new robust heterogenous photoredox catalytic systems. The photocatalytic activity and recyclability were evaluated through the model aza-Henry reaction. Then, a comparative study has been run on reductive cyclization reaction to estimate the impact of non-covalent interactions brought by the porous material to the selectivity of the reaction (Scheme 1b).

## 2. Results and Discussion

### 2.1. Preparation of Hybrid Photocatalysts

**Scheme 2:**
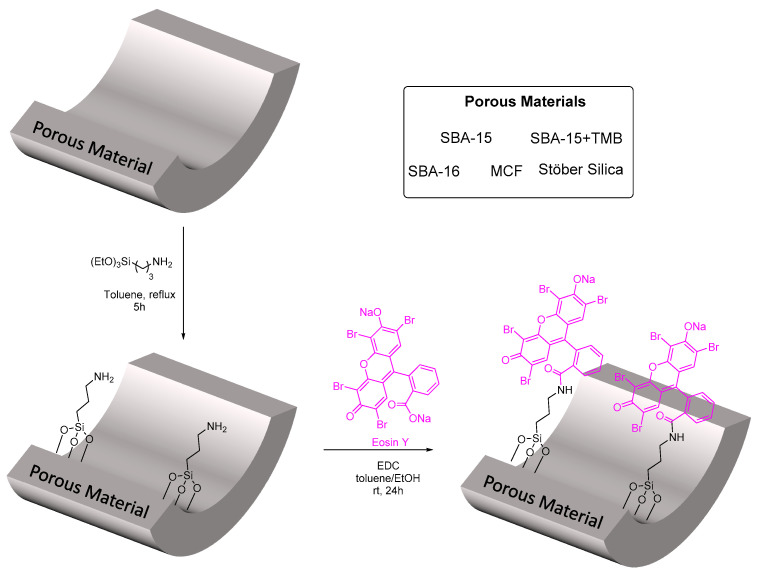
Preparative sequence of hybrid photoredox catalysts.

Our strategy relies on the functionalization of porous silica materials according to a two-step procedure (Scheme 2). First, amino-functionalization of the material was achieved by grafting 3-aminopropyltriethoxysilane (APTES) on free silanol groups. Then, the covalent linkage of EY to the porous material was realized through the formation of strong amide bond between the carboxylic groups of the Eosin Y and the amino groups of the APTES moiety by the EDC (1-ethyl-3-(3-dimethylaminopropyl)carbodiimide) coupling activation. After the second post-synthetic step, the color of the modified porous materials changes clearly from white into fuchsia.

The optimization of the grafting was first realized on SBA-15, and extended to other materials: SBA-16, MCF (mesocellular foam), Stöber Silica and SBA-15+TMB. For MCF and SBA-15+TMB, we modified the classical procedure of SBA-15 by adding trimethylbenzene (TMB/P123 = 0.6 for MCF and TMB/P123 = 0.1 for SBA-15+TMB). For SBA-15+TMB the same morphology as SBA-15 was obtained but with a larger pore diameter. To control the porosity effect during the photocatalysis, we prepared a non-porous material (dense spherical particles) the Stöber silica.

The covalent bonding and the Eosin Y loading have been verified through complementary technics. From the IR spectra of functionalized samples, C-H band of stretching vibrations were observed between 2935 and 2945 cm^−1^ as well as NH_2_ bending vibration around 1550 cm^−1^ (see Supplementary Materials Figure S3). This confirmed the presence of an amino group and therefore the functionalization of materials with APTES. The Eosin Y functionalization of the materials was also confirmed by the presence of specific aromatic elongation band C=C in the 1454 and 1500 cm^−1^ range except for SBA-16-APTES-EY and Stöber-APTES-EY because the loading of EY was very low. The XRD patterns of porous material (PM), PM-APTES and PM-APTES-EY have been recorded (see Supplementary Materials Figure S2). For each hybrid photocatalyst, the functionalized and grafted samples exhibit intense peaks attesting that PM retain the ordered mesoporous structure after each post modification. The X-ray diffractograms of the MCF materials do not show any peak since the MCF material is considered as mesocellular foam with very large pores (22 nm). This organization cannot be observed by this technique. In all the cases, the BET surface area, the diameter of the pores as well as the volume of the mesopores decrease after functionalization and grafting (Table 1), which confirms the grafting of the major part of the amino groups by Eosin Y on the pore surface. For the Stöber silica, which are dense 308 nm nm-diameter spheres (see Supplementary Materials Table S1 and Figure S5), the S_BET_ was not impacted by the functionalization steps, validating the outer surface modification compared to the inner pores functionalization with SBA-15, SBA-15-TMB, MCF and SBA-16. For SBA-16-APTES-EY, such information cannot be obtained due to the blocked porosity related to the cage-like morphology of its pores. The estimation of EY loading (wt%) have been achieved using TGA (ThermoGravimetric Analysis), which is a method able to measure the weight loss of a sample when heated. Thus, the comparison between APTES-grafted and Eosin Y-APTES grafted material allowed to determine the Eosin Y loading. We found for this series of photocatalyst, that EY loading was measured between 8.7 and 1.6 wt% and these values were used for the evaluation of photocatalytic activity (Table 1).

### 2.2. Photoredox Aza-Henry Reaction

The aza-Henry reaction is a relevant organic transformation which aims to create C-C bond under oxidative conditions. We focused on the dehydrogenative cross-coupling reaction between 2-phenyl-1,2,3,4-tetrahydroisoquinoline **1** and nitromethane (Table 2). First, we compare the catalytic property of the new SBA-15-APTES-EY, free EY and SBA-15-APTES-RB [34].

Based on previously developed experimental conditions [34], we confirmed that 5 mol% of Eosin Y in nitromethane as solvent under green light irradiation (562 nm) allowed to obtain compound **2** after 24 h in 96% yield (Table 2, entry 1). With the same conditions, the new SBA-15-APTES-EY catalyst proved to be superior to SBA-15-APTES-RB, allowing to generate **2** in 97% compared to 85% yield obtained when using SBA-15-APTES-RB (Table 2, entries 2–3). To secure that the catalytic activity came from the whole hybrid system, SBA-15 and SBA-15-APTES were engaged, and no reaction occurred (Table 2, entries 4–5). Green light irradiation was also crucial for the transformation while no reaction occurred when the reaction was run in the dark (Table 2, entry 6). The optimization of the reaction time was done and reduced to 16 h and 8 h. In 16 h, no significant decrease in the yield was observed while a lower yield of 80% was obtained in 8 h (Table 2, entries 7–8). With free Eosin Y, this modification did not impact the reaction (Table 2, entries 9–10). To improve the sustainability of the reaction process, we change the nitromethane as solvent to the more environmentally benign isopropanol or water, keeping nitromethane as reagent (10 equivalents). In isopropanol, the aza-Henry alkylation was still very efficient in 16 h and **2** was obtained in 94% yield (Table 2, entry 11). In water, the reaction was slower but still productive, affording **2** in 20% and 34% after 24 h and 48 h, respectively (Table 2, entries 12–13). To better evaluate the efficiency of the hybrid photocatalyst, loadings were decreased successively to 1 and 0.5 mol%. The heterogeneous SBA-15-APTES-EY tended to have a similar activity at 1 mol% with 92% yield and a slight erosion of the yield at 0.5 mol% (Table 2, entries 14–15). This set of optimization demonstrates the high efficiency of this new heterogeneous photoredox catalytic system to promote the aza-Henry reaction.

**Figure 1:**
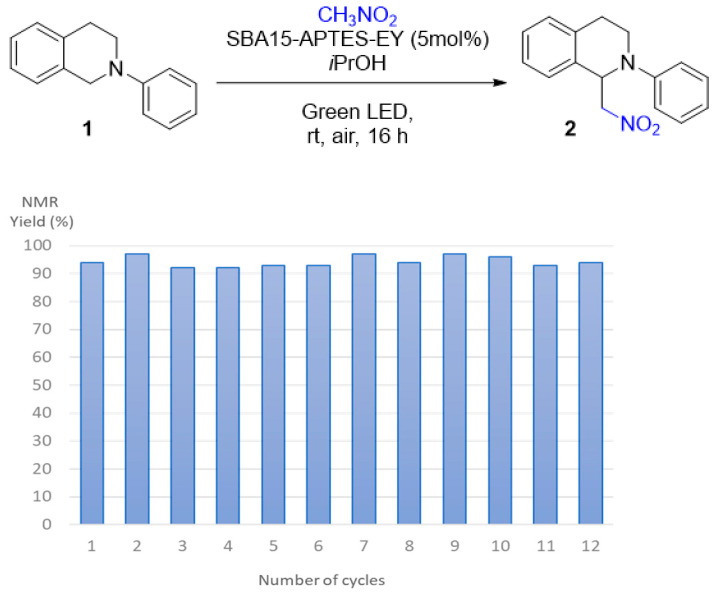
Photocatalyst recycling for aza-Henry reaction.

Then, recyclability was tested at room temperature, under green LED irradiation, using iso-propanol (*i*PrOH) as a green solvent and a reaction time of 16 h (Figure 1). Although a catalyst loading of 1 mol% leads to almost full conversion of our starting material **1**, the catalyst loading for recycling was set at 5 mol%, because dealing with 1 mol% of the catalyst, while fixing the amount of starting material (0.1 mmol), would be pretty difficult in terms of practical work during the recycling process. After each reaction cycle, the heterogeneous catalyst was recovered by centrifugation and dried under vacuum to reuse in a new cycle. Excellent performance of SBA-15-APTES-EY hybrid photocatalyst was observed without any loss in activity for twelve consecutive cycles. This clearly demonstrated the robustness of this photoredox catalyst. It is still highly active after 12 cycles and did not need any regeneration step between each cycle. This catalytic system appeared more robust compared to the Rose Bengal analog, which showed significant decrease in its activity after 8 cycles [34].

### 2.3. Impact of the Support on the Photoreductive Cyclization of Aryl Bis(Enones)

**Scheme 3:**
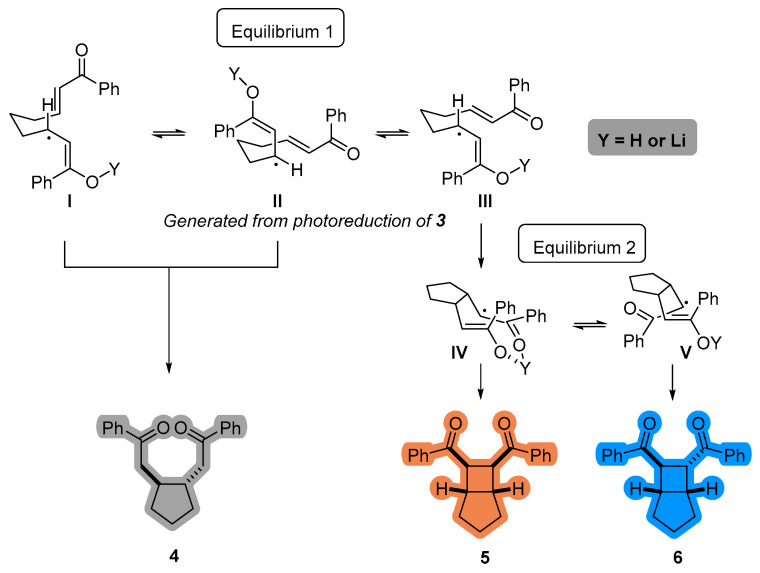
Proposed mechanistic pathways.

To push forward the investigation, we decided to evaluate the impact of such system on the chemical selectivity issue. For this purpose, the [2 + 2] cycloaddition of aryl enones, and particularly bis(enone) substrates, would help us to gain insights in the intrinsic reactivity of the series of hybrid photoredox catalysts. In confined environment, thermodynamic and kinetic parameters can be modified through the stabilization of intermediates by non-covalent interactions as well as the local polarity or the reaction entropy (mobility of molecules) [35,36,37,38,39,40,41,42,43,44,45,46]. The shielding effect can protect some very reactive intermediates (carbocations, radicals, excited states …) due to the confined environment. More generally, electron transfer and radical catalysis are strongly impacted in cage cavities. The selectivity is also altered in constrained media by avoiding competitive pathways. This aspect of confined environment has been extensively studied for applications through organic photochemical transformations and highlight the benefit of such environments. Bis(enone) compounds are substrates of choice for model transformations because they proved to be very sensitive to the cyclization conditions, including physical and chemical influences [47,48,49,50]. Indeed, the environment acidity plays a crucial role in the behavior of this transformation. As reported by Yoon [47,48], Lewis and Brønsted acidities provide divergent selectivities for the cycloaddition reaction. Depending on reaction conditions radical anion or neutral radical are generated by photoreduction of bis(enone) **3.** According to the Beckwith–Houk model (Scheme 3) [51], three main conformers are in equilibrium (Equilibrium 1). **I** and **II** give the *trans*-diketone **4** after 5-exo-trig cyclization and H-abstraction. However, **III** can also undergo a 5-*exo*-trig cyclization to provide **IV** in equilibrium with **V** (Equilibrium 2) which, respectively give the products **5** and **6**. Each parameter that could influence the equilibrium 1 and 2, should provide difference between the ratio of products **4**, **5**, **6**. In our case, we hypothesize that the porous material could impact selectivity of the reaction by changing the energy of radical intermediates through non-covalent interactions as well as the conformer populations.

**Figure 2:**
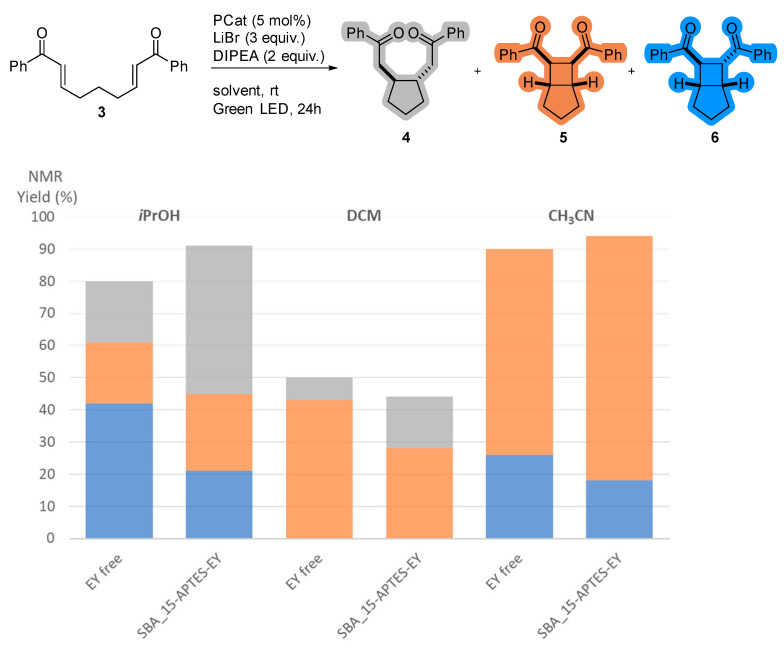
Influence of solvent on photoreductive cyclization of **3**.

To highlight this effect, we used the modified Yoon’s conditions [47]: 1 equivalent of **3**, 5 mol% of hybrid photocatalyst with 2 equivalents of diisopropylethyl amine as reductive quench and 3 equivalents of LiBr as Lewis Acid. First, we decided to investigate the solvent effect in this reaction. We compared the selectivity between free-EY and SBA-APTES-EY in *i*PrOH, dichloromethane (DCM), and acetonitrile (CH_3_CN) (Figure 2).

In *i*PrOH, Eosin Y gave the products mixture in 80% yield whereas SBA-15-APTES-EY proved to be more efficient with 92%. Interestingly, the products distribution is slightly different with **4**:**5**:**6** 19%:19%:42% for Eosin Y and 46%:24%:21% for SBA-15-APTES-EY. In DCM, the compounds distributions were less contrasted. With Eosin Y (50% yield) and SBA-15-APTES-EY (44% yield) allowed to form **5** as major product with minor **4**. The ratio **4:5** was 43%:7% for free Eosin Y and 28%:16% for SBA-15-APTES-EY. In CH_3_CN, the chemical yields of the reactions were very high compared to precedent reactions in DCM and *i*PrOH. The selectivities were almost similar between catalytic systems. Indeed, compound **4** was not observed in this solvent, independently of the catalyst. Eosin Y and SBA-15-APTES-EY provided **5**:**6** mixtures in 90% and 94% yield, respectively and **5** was the major product (64%:26% vs. 76%:18%). Due to the polarity and protic character of solvent, we observed a strong solvent effect that play on the distribution of products. We also detected tiny but significant variations between the ratio of products when homogeneous and heterogeneous catalysis were compared. This last observation suggest that the porous material is not innocent in the reaction.

**Figure 3:**
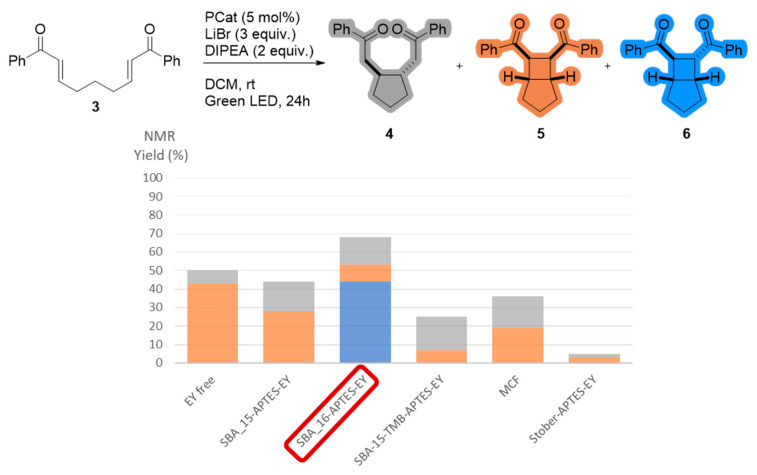
Influence of porous material on photoreductive cyclization of **3** in presence of LiBr.

In a next series of experiments, the influence of the material was investigated for the cycloaddition of bis(enone) 3 (Figure 3). Four new materials SBA-16, SBA-15-TMB, MCF, Stöber silica were compared to precedent catalysts Eosin Y and SBA-15 in dichloromethane. Because the reaction is strongly influence by Lewis and Brønsted acidity we ran two sets of reactions in presence or absence of LiBr and with N, N-Diisopropylethylamine (DIPEA) as a co-reductant. For all the experiments, reactions were repeated twice, an average of product repartitions is presented.

**Figure 4:**
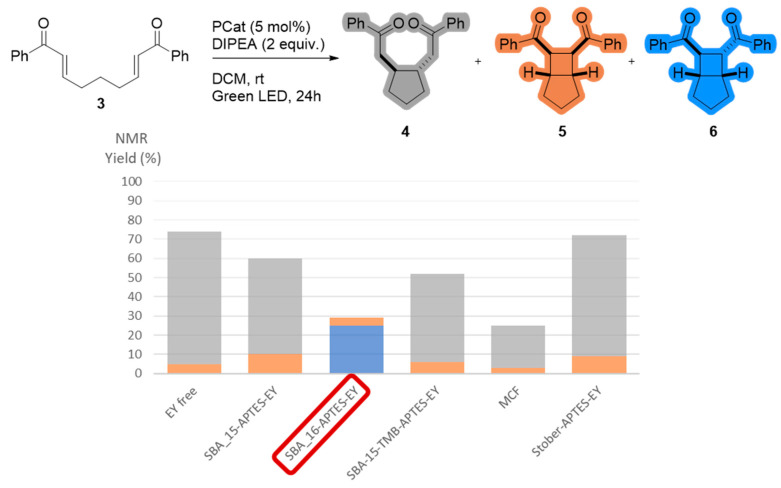
Influence of porous material on photoreductive cyclization of **3** in absence of LiBr.

In presence of LiBr, **5** is obtained as the major product with EY-free, SBA-15-APTES-EY, SBA-15-TMB-EY, MCF (Figure 3). The reaction is not efficient in presence of Stöber-APTES-EY catalyst, only few traces of products have been detected. Interestingly, the selectivity changes completely when SBA-16-APTES-EY is used. In this case, the reaction is slightly more efficient and **6** is observed as the main product (ratio **4**:**5:6** = 9%:15%:44%). This result proves that the support of the heterogeneous photocatalyst is not innocent and can influence the conformational equilibrium between reactive intermediates changing the selectivity of the reaction. Without Lewis acid (LiBr free), the homogeneous reaction with EY gives essentially the product **4** in 69% yield (Figure 4). The same tendency was observed with SBA-15-APTES-EY, SBA-15-TMB-APTES-EY, Stöber-APTES-EY. The reaction with MCF-APTES-EY was less efficient but similar selectivity was obtained (**4** in 22% yield). Once again, the SBA-16-APTES-EY revealed to be the most interesting result since a reverse selectivity was measured with the predominance of compound **6** (ratio **4**:**5**:**6** = 0%:4%:22%). To verify the stability of **5** and its possible isomerization (**5** to **6**), we ran a control experiment (see Supplementary Materials Figure S7). We exposed **5** under green LED irradiation in presence of SBA16-APTES-EY, and we were pleased to observe the photostability of the compound because no trace of isomerization was detected. Thus, the singular selectivity with SBA-16-APTES-EY can only be attributed to the inorganic part of the photocatalyst. SBA-16 is composed of “Cage-like” mesoporous structure and after functionalization the porosity is blocked (Table 1, entry 2). The reaction should occur on the surface, at the entrance of the pore, and specific interactions between the reactive intermediates and the material favor the conformer **III** and **V** in the process explaining the formation of **6**.

## 3. Materials and Methods

**Materials.** Eosin Yellowish (Eosin Y), N, N-Diisopropylethylamine (DIPEA) and Potassium phosphate anhydrous, 97% were purchased from Alfa Aesar. P123, TEOS 98%, F127, Ammonium hydroxide aqueous solution (30–33% *w*/*w*) and Copper(I) iodide (purum ≥ 99.5%) were purchased from Sigma-Aldrich. EDC and Iodobenzene was purchased from Acros Organics and FluoroChem, respectively. HCl 37% and APTES 98% were purchased from Carlo Erba and Abcr, respectively. 1,2,3,4-Tetrahydroisoquinoline was purchased from TCI. Ethylene glycol was purchased from Riedel-de Haën, Honeywell. All solvents were purchased from VWR.

Preparation of SBA-15. As reported by Zhao et al., a typical synthesis of SBA-15 is realized [52]. A measure of 2 g of Pluronic^®^ P123 (EO_40_PO_70_EO_40_with EO = ethylene oxide and PO = propylene oxide) was dissolved in aqueous solution of HCl obtained from a mixture of 64 g of distilled water and 11 g of 37 wt% HCl. The dissolution was carried out for 1 h in a water bath at 40 °C while stirring at a speed of 500 rpm. After the dissolution of P123, 4.3 g of tetraethyl orthosilicate (TEOS) were added slowly to the reactor using a peristaltic pump operated at 8 mL/min. The mixture was stirred for further 5 min at a speed of 500 rpm and kept in water bath at 40 °C for 2 h. After that, the mixture placed in a closed flask was held in the oven at 90 °C for 24 h, and then allowed to cool down. The precipitated solid was recovered by filtration on a Büchner, then washed abundantly with demineralized water and then dried in oven at 70 °C overnight. The obtained solid was calcined in air to remove the template by heating from room temperature to 300 °C in 6 h and maintained at this temperature for 4 h.

Preparation of SBA-16. Under acidic condition using Pluronic^®^ F127 (EO_106_PO_70_EO_106_) as a porogen agent SBA-16 was prepared [53]. A measure of 2 g of F127 was dispersed in acidic solution prepared by adding 146 mL of deionized (DI) water to 22 mL of 37 wt% HCl at 60 °C while stirring at a speed of 500 rpm. Subsequently, 9.9 g of tetraethyl orthosilicate (TEOS) was added onto the solution at fixed rate 8 mL/min and stirring continued for 10 min. The reaction mixture was kept at 60 °C for 2 h. Then, the reaction mixture was removed from the water bath and placed in oven at 80 °C for 24 h The solid product was recovered and washed with demineralized water and then dried in oven at 70 °C overnight. The obtained solid was calcined in air to remove the template by heating from room temperature to 300 °C in 6 h and maintained at this temperature for 4 h.

Preparation of SBA-15+TMB: 7 g of P123 was dissolved in 39.2 g of HCl diluted with 223.5 g of distilled water. Trimethylbenzene (TMB) was added such as molar ratio TMB/P123 = 0.1. The solution was stirred at 40 °C for 1 h. Then, 14.86 g of TEOS was added to the solution at fixed rate 8 mL/min and stirring was continued for 10 min. The reaction mixture was kept at 40 °C for 2 h. The solution was placed in an oven at 90 °C for 24 h. Then, the solid was collected by filtration, washed by distilled water and dried at 70 °C overnight. The calcination was carried out at 300 °C for 6 h [54].

Preparation of MCF: The MCF, which is mesocellular foam was prepared like an SBA-15 except for the addition of a swelling agent the trimethylbenzene (TMB) as reported elsewhere [54]. Thus, 7 g of P123 template was dissolved in a dilute hydrochloric acid solution (39.2 g HCl + 223.5 g water). Then, 0.7 g of TMB (molar ratio TMB/P123 = 0.6) was added. The mixture was stirred at a speed of 500 rpm, under 40 °C for 1 h. The TEOS (14.86 g) was then added slowly (8 mL/min). Then, the mixture was left at 40 °C for 2 h, then aged at 90 °C for 24 h. The material was filtered and washed with distilled water then dried at 70 °C. Removal of the template was achieved by calcination at 300 °C for 6 h.

Preparation of Stöber silica. The preparation of Stöber silica is made as described by Li Shanshan et al. [55]. First, 9 mL of ammonia was mixed with 27.5 mL of deionized water and 199 mL of ethanol. The mixture was stirred at room temperature for 30 min. A measure of 14.5 mL of TEOS was speedily added to mixture and the agitation is kept for 1 h. Afterwards, a centrifugation is made at 10,000 rpm to recover the precipitate of silica. Five cycles of washing with demineralized water were realized and then the sample is dried for 2 h in oven at 120 °C.

Coupling of Eosin Y. The immobilization of Eosin Y was performed by a covalent link using a two-step post-synthetic strategy [34].

The functionalization of materials: 1 g of material (SBA-15 or SBA-16 or Stöber silica or MCF) was charged in a round bottom flask and stirred under vacuum while heating at 100 °C to remove water or moisture. Furthermore, 15 mL of toluene and 1.0 mL of 3-aminopropyltriethoxysilane (APTES) were added under N_2_ atmosphere. The reaction was set on a reflux apparatus at 110–115 °C for 5 h. The obtained solid was washed with EtOH and centrifuged 5 times then dried overnight in the oven at 70 °C.

The coupling of EY with grafted materials: 1 g of EY and 1-ethyl-3-(3-dimethylaminopropyl)carbodiimide (EDC, 3 equiv.) were dissolved in 15 mL of ethanol, and the mixture was stirred in the absence of light for half an hour in order to activate the EDC. After that, 1 g of the APTES-modified solid was added to the mixture, where it was allowed to be stirred in the absence of light. After 24 h, the reaction was quenched with 10 mL of water to deactivate the unreactive EDC. The obtained solid was washed several times with ethanol and centrifuged. Then, it was dried under vacuum.

Characterization methods. X-ray diffraction (XRD) measurements were recorded using a Malvern Panalytical X’Pert Pro diffractometer (Malvern Panalytical; Palaiseau, France) operating with Cu Kα radiation. The powder 017 pattern was collected at 22 °C in the ranges of 0.5 < 2θ < 10°, with a 2θ angle step of 0° and a time step of 300 s. Nitrogen adsorption−desorption isotherms were performed at −196 °C using a Micromeritics TRISTAR equipment (Micromeritics, Merignac, France). Prior to the adsorption measurements, the samples were outgassed at 70 °C overnight under vacuum to eliminate physisorbed water. Low degassing temperature was chosen to avoid the organic material decomposition process. The specific surface area (S_BET_), microporous volume (Vmicro) and mesopore size distributions were calculated by the BET, t-plot and BJH (Barrett-Joyner-Halenda) on the desorption branch methods, respectively. The mesoporous volume was determined by the subtraction of the microporous volume from the total porous volume. SEM analyses were recorded on JSM-7900F (JEOL), the images were made with an acceleration voltage of 2 KV. using the images given by this characterization method, an average particle size (10 measurements minimum for each material) is made based on the scale of each figure. FT-IR spectra were recorded from pellets (1%) in KBr on a Bruker Equinox IFS55 spectrometer equipped with a pyroelectric detector (DTGS type) with a resolution of 4 cm^−1^. Thermogravimetric Analyses (TGA) were carried out on a TG METTLER TOLEDO STAR apparatus (METTLER TOLEDO, Viroflay, France), under air flow, with a heating rate of 5 °C/min from 30 to 800 °C. UV-Vis absorption spectra were recorded using a Molecular Devices Spectra Max iD_3_ multi-mode microplate reader. The absorption measurements are recorded in the wavelength of the range of 400 and 800 nm with a step of 5 nm.

### 3.1. Aza Henry Photocatalyzed Reaction 

Preparation of 2-N-phenyl-1,2,3,4-tetrahydroisoquinoline 1: Copper(I) iodide (200 mg) and potassium phosphate (4.25 g, 20.0 mmol) were put into a Schlenk tube. The tube was evacuated and back filled with nitrogen. 2-Propanol (10.0 mL), ethylene glycol (1.11 mL, 20.0 mmol), 1,2,3,4-tetrahydro-isoquinoline (2.0 mL, 15 mmol) and iodobenzene (1.12 mL, 10.0 mmol) were added successively by micro-syringe at room temperature. The reaction mixture was heated at 85–90 °C and kept for 24 h and then allowed to cool to room temperature. Diethyl ether (20 mL) and water (20 mL) were then added to the reaction mixture. The organic layer was extracted by diethyl ether (2 × 20 mL). The combined organic phases were washed with brine and dried over magnesium sulphate. The solvent was removed by rotary evaporation the product was purified by column chromatography on silica gel (petroleum ether; petroleum ether/ethyl acetate = 95/5) to give 1 in 67% yield. Light yellow oil. ^1^H NMR (400 MHz, CDCl_3_) δ 7.33–7.28 (m, 2H), 7.21–7.15 (m, 4H), 7.00 (dd, *J* = 8.8, 0.9 Hz, 2H), 6.89–6.79 (tt, *J* = 7.3, 1.0 Hz, 1H), 4.42 (s, 2H), 3.58 (t, *J* = 5.9 Hz, 2H), 3.00 (t, *J* = 5.8 Hz, 2H). ^13^C NMR (100 MHz, CDCl3) δ 150.7, 135.0, 134.6, 129.3 (2C), 128.6, 126.7, 126.5, 126.2, 118.8, 115.3 (2C), 50.9, 46.6, 29.3.

Photocatalyzed Aza-Henry reaction with the hybrid heterogeneous catalysts: First, 2-N-phenyl-1,2,3,4-tetrahydroisoquinoline 1 (0.1 mmol), the catalyst (with various EY loading) and either 1 mL or 10 equiv. (1 mmol) of nitromethane with 1 mL of the appropriate solvent (if applicable) were charged in a reaction glass tube with magnetic stirring bar. The reaction mixture was stirred under green LED (565 nm) irradiation (approximately 7 cm away from the LED lamp) at room temperature for different reaction times. The setup of the whole reaction was covered with aluminum foil. After the reaction, the solvent was evaporated under vacuum and the crude product was directly loaded onto a short silica gel column. The filtration was performed using petroleum ether/ethyl acetate (7/3). After removing solvent, the reaction crude was obtained as yellow oil. The product was quantified by 1H-NMR, with 1,3,5-trimethoxybencene as internal standard.

1-(Nitromethyl)-2-phenyl-1,2,3,4-tetrahydroisoquinoline 2 [34]: yellow oil. ^1^H NMR (400 MHz, CDCl_3_) 7.33–7.10 (m, 6H), 6.99 (d, *J* = 8.6 Hz, 2H), 6.86 (td, *J* = 7.3, 0.7 Hz, 1H), 5.56 (t, *J* = 7.2 Hz, 1H), 4.88 (dd, *J* = 11.8, 7.8 Hz, 1H), 4.57 (dd, *J* = 11.8, 6.6 Hz, 1H), 3.77–3.47 (m, 2H), 3.17–2.95 (m, 1H), 2.80 (dt, *J* = 16.3, 4.9 Hz, 1H).

Preparation of (2E,7E)-Diphenylnona-2,7-diene-1,9-dione 3 [47,48,49,50]: 1 equiv of Bromide (2-bromoacetophenone) was added gradually to a solution of PPh_3_ (1equiv, 0.3 M) in toluene. The mixture was stirred overnight at room temperature and then filtered on Büchner and washed with toluene (2 × 100 mL) and hexane (2 × 100 mL) to give the phosphonium salt. For 2 g of Phosphonium bromide salt dissociated in a solution of 50 mL methanol and 50 mL water, sodium hydroxide (1.5 M) was added up to get a pH value of the reaction of 8. The reaction was stirred at room temperature overnight. The extraction of reaction was done after evaporation of methanol and using dichloromethane (3 × 40 mL) and water (20 mL). Combined organic phases were dried over MgSO_4_ and then phosphorus ylide was obtained. A measure of 1 equiv of ylide was dissolved in THF and mixed with glutaric dialdehyde solution (0.3 equiv) and MgSO_4_ (ca. 30 g). The mixture was stirred at room temperature for 2 days. Then, the sample was filtered and the reaction solvent was evaporated. Silica gel chromatography (40–60% ether in petrol) was used to purify the bis(enone) 3 (32% yield). yellow oil. ^1^H NMR (400 MHz, CDCl_3_) 7,93 (dt, *J* = 8.5, 1.7 Hz, 4H), 7.59–7.53 (m, 2H), 7.51–7.43 (m, 4H), 7.07 (dt, *J* = 15.3, 6.8 Hz, 2H), 6.92 (dt, *J* = 15.4, 1.3 Hz, 2H), 2.4 (td, *J* = 8.0, 1.2 Hz, 4H), 1.87–1.73 (m, 2H).^13^C NMR (101 MHz, CDCl_3_) δ 190.6, 148.5, 137.8, 132.7, 128.5, 128.5, 126.5, 32.2, 26.7.

### 3.2. Photocatalyzed Cycloaddition of 3

In a schlenk tube, (2E, 7E) -Diphenylnona-2,7-diene-1,9-dione (0.07 mmol) was mixed with 5 mol% of the photocatalyst with N, N-Diisopropylethylamine (2 equiv) or in the presence or absence of LiBr (3 equiv). A measure of 0.7 mL of the degassed solvent were added under nitrogen then the reaction medium was stirred by ultrasound. the mixture was irradiated by a green LED. The setup of the whole reaction was covered with aluminum foil while continuing the agitation for 24 h. After the reaction, the solvent was evaporated under vacuum. The products obtained by the cycloaddition reaction were quantified by ^1^H NMR (NMR yield) using 1 equiv. of diphenylethylene as an internal standard.

Trans-2,2′-(Cyclopentane-1,2-diyl)bis(1-phenylethanone) 4: ^1^H NMR (500 MHz, CDCl3) δ 7.94 (dt, *J* = 8.5, 1.7 Hz, 4H), 7.54 (tt, *J* = 7.3, 1.3 Hz, 2H), 7.44 (t, *J* = 7.9 Hz, 4H), 3.20 (dd, *J* = 16.5, 4.3 Hz, 2H), 2.94 (dd, *J* = 16.5, 8.2 Hz, 2H), 2.19 (m, 2H), 1.99 (m, 2H), 1.63 (m, 2H), 1.28 (m, 2H); ^13^C NMR (125 MHz, CDCl_3_) 200.2, 137.2, 132.9, 128.6, 128.1, 44.0, 41.6, 32.5, 23.7.

Cis-Bicyclo [3.2.0]heptane-6,7-diylbis(phenylmethanone) 5: ^1^H NMR (400 MHz, CDCl_3_) 7.85–7.66 (m, 4H), 7.45(tt, *J* = 7.4 Hz, 2H), 7.39–7.31 (m, 4H), 3.86 (d, *J* = 4.0 Hz, 2H), 3.25–3.15 (m, 2H), 2.09–1.99 (m, 2H), 1.90–1.81 (m, 2H), 1.76–1.60 (m, 2H). ^13^C NMR (500 MHz, CDCl_3_) δ 198.6, 136.4, 132.5, 128.5, 127.8, 48.3, 39.1, 32.5, 25.2.

Trans-Bicyclo [3.2.0]heptane-6,7-diylbis(phenylmethanone) 6: ^1^H NMR (300 MHz, CDCl_3_) δ 8.02 (m, 2H), 7.94 (m, 2H), 7.55 (m, 2H), 7.45 (m, 4H), 4.57 (dd, *J* = 10.3, 7.9 Hz, 1H), 4.29 (m, 1H), 3.24 (m, 1H), 3.06 (q, *J* = 6.9 Hz, 1H), 1.84 (m, 3H), 1.53 (m, 1H), 1.42 (m, 2H). ^13^C NMR (75 MHz, CDCl_3_) δ 200.3, 198.1, 136.2, 135.7, 133.2, 133.1, 128.8, 128.6, 128.3, 128.1, 43.1, 43.0, 40.5, 40.4, 32.1, 28.5, 25.6.

## 4. Conclusions

The heterogenization of the homogeneous photocatalyst Eosin Y, on the APTES modified mesoporous silica produced a robust organic/inorganic hybrid heterogeneous photocatalyst. These new photocatalytic systems have been successfully prepared, characterized and utilized in aza-Henry and cycloaddition reactions. Taking advantage of the heterogeneous catalysts, 12 cycles have been realized with the same catalyst to perform aza-Henry reaction without alteration of the photocatalytic activity. Finally, we have demonstrated that the porous inorganic support is not spectator during the transformation. Due to the non-covalent interactions between the substrate (aryl bis(enone)) and the mesoporous material contained in SBA-16-APTES-EY, the regio/stereoselectivity is affected, inversing completely the ratio of products observed in homogeneous photocatalysis.

## Figures and Tables

**Table 1:** Textural properties of heterogeneous photocatalysts and EY loading.

Entries	Photocatalyst	Mesostructure	S_BET_ (m^2^/g)	Pore Diameter (nm)	Mesoporous Volume (cm^3^/g)	EY wt%
1	SBA-15-APTES-EY	hexagonal 2D	202	6.5	0.31	5.7
2	SBA-16-APTES-EY	cubic body-centered	6	-	-	2.0
3	SBA-15+TMB-APTES-EY	hexagonal 2D	223	7.5	0.40	6.4
4	MCF-APTES-EY	mesocellular foam	195	20	0.50	8.7
5	Stöber-APTES-EY	-	12	-	-	1.6

**Table 2:** Photocatalyzed aza-Henry reaction.

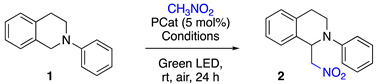				
Entries	PCat	Solvent	Deviation from Standard Conditions	Yield (%) ^1^
1	Eosin Y	CH_3_NO_2_	-	96
2	SBA-15-APTES-EY	CH_3_NO_2_	-	97
3	SBA-15-APTES-RB	CH_3_NO_2_	-	85
4	SBA_15	CH_3_NO_2_	-	0
5	SBA-15-APTES	CH_3_NO_2_	-	0
6	SBA-15-APTES-EY	CH_3_NO_2_	No light	0
7	SBA-15-APTES-EY	CH_3_NO_2_	16 h	97
8	SBA-15-APTES-EY	CH_3_NO_2_	8 h	80
9	Eosin Y	CH_3_NO_2_	16 h	96
10	Eosin Y	CH_3_NO_2_	8 h	98
11	SBA-15-APTES-EY	iPrOH	CH_3_NO_2_ (10 equiv.), 16 h	94
12	SBA-15-APTES-EY	Water	CH_3_NO_2_ (10 equiv.), 24 h	20
13	SBA-15-APTES-EY	Water	CH_3_NO_2_ (10 equiv.), 48 h	34
14	SBA-15-APTES-EY	iPrOH	CH_3_NO_2_ (10 equiv.), 16 h, PCat (1 mol%)	92
15	SBA-15-APTES-EY	iPrOH	CH_3_NO_2_ (10 equiv.), 16 h, PCat (0.5 mol%)	77

^1 1^H NMR yield determined with an internal standard 1,3,5-trimethoxybenzene.

## Data Availability

Original data is not avaible due to on-going study.

## Supplementary Materials

The following supporting information can be downloaded at: https://www.mdpi.com/article/10.3390/molecules28020549/s1, Figure S1. XRD patterns of (A) SBA-15, SBA_15-APTES, SBA-15-APTES-EY; (B) SBA-16, SBA-16-APTES, SBA-16-APTES-EY; (C) Stöber, Stöber-APTES, Stöber-APTES-EY; (D) SBA-15+TMB,.SBA-15+TMB -APTES, SBA-15+TMB -APTES-EY; (E) MCF, MCF-APTES , MCF-APTES-EY, Figure S2. FT-IR followed the preparative sequence of hybrid photoredox catalysts of (A) SBA-15, SBA_15-APTES, SBA-15-APTES-EY; (B) SBA-16, SBA-16-APTES, SBA-16-APTES-EY; (C) Stöber, Stöber-APTES, Stöber-APTES-EY; (D) SBA-15+TMB, SBA-15+TMB -APTES, SBA-15+TMB -APTES-EY; (E) MCF, MCF-APTES, MCF-APTES-EY. Figure S3. N2 physisorption isotherms. (A) SBA-15, SBA_15-APTES, SBA-15-APTES-EY; (B) SBA-16, SBA-16-APTES, SBA-16-APTES-EY; (C) Stöber, Stöber-APTES, Stöber-APTES-EY; (D) SBA-15+TMB, SBA-15+TMB -APTES, SBA-15+TMB -APTES-EY; (E) MCF, MCF-APTES, MCF-APTES-EY. Figure S4. The temperature range which corresponds to the elimination of surfactant (from ~170 to 300 °C) is different from that of eosin (from ~400 to 600 °C). (A) SBA-15, SBA_15-APTES, SBA-15-APTES-EY; (B) SBA-16, SBA-16-APTES, SBA-16-APTES-EY; (C) Stöber, Stöber-APTES, Stöber-APTES-EY; (D) SBA-15+TMB, SBA-15+TMB -APTES, SBA-15+TMB -APTES-EY; (E) MCF, MCF-APTES, MCF-APTES-EY, (F) SBA-15, As-SBA-15. Figure S5. Photocatalysts: (1) EOSIN Y, (2) MCF-APTES-EY, (3) SBA-15+TMB-APTES-EY, (4) SBA-15-APTES-EY, (5) SBA-16-APTES-EY, (6) Stöber-APTES-EY. Figure S6. (a-b) LED was placed at 5 cm to the reaction tube. (c) M565L3, light-emitting diode (LED, nominal wavelength: 565 nm, output power: 979 mW, irradiance: 11.7 μW/mm^2^). Figure S7. Stability of (5) under reaction conditions with SBA-16-APTES-EY. Figure S8. 1H NMR of the crude reaction, the starting material 5 was recovered untouched after 16 h of irradiation. Figure S9. 1H and 13C of 2-N-phenyl-1,2,3,4-tetrahydroisoquinoline (1) [34] (a) 1H-NMR; (b) 13C-NMR. Figure S10. 1H and 13C of 1-(Nitromethyl)-2-phenyl-1,2,3,4-tetrahydroisoquinoline (2) [34]. (a) 1H-NMR (b) 13C-NMR. Figure S11. 1H of (2E,7E)-Diphenylnona-2,7-diene-1,9-dione (3) [49]. (a) 1H-NMR (b) 13C-NMR. Figure S12. 1H of Cis-Bicyclo[3.2.0]heptane-6,7-diylbis(phenylmethanone (5) [57]. (a) 1H-NMR (b) 13C-NMR. Figure S13. 1H NMR of inseparable mixture of Trans-2,2'-(Cyclopentane-1,2-diyl)bis (1-phenylethanone) [48] and Trans--Bicyclo[3.2.0]heptane-6,7-diylbis(phenylmethanone) (6) [50]. Table S1. Particle sizes measurement. Table S2. Summary of the textural properties of materials., Table S3. Estimation of the EY loading by TGA (wt% A) and UV-Vsible spectroscopy (wt% B).; References [34,48,49,50,56,57] are cited in the supplementary materials.

## Sample Availability

Samples of the compounds are not available from the authors.
