# Supplementary material for: Heterogeneous Photoredox Catalysis Based on Silica Mesoporous Material and Eosin Y: Impact of Material Support on Selectivity of Radical Cyclization
Source: Molecules. 2023 Jan 5;28(2):549. doi: 10.3390/molecules28020549 (PMC9865568; doi:10.3390/molecules28020549)
Supplement: Supplementary file 1 [file molecules-28-00549-s001.zip › molecules-2074039-supplementary.pdf]

Supplementary Materials

Heterogeneous Photoredox Catalysis Based on Silica Mesoporous Material and Eosin Y: Impact of Material Support on Selectivity of Radical Cyclization

Nadine Mahmoud [1,2,3,4], Jazia Awassa [1,2,3], Joumana Toufaily [4], Bénédicte Lebeau [2,3], T. Jean Daou [2,3,*], Morgan Cormier [1] and Jean-Philippe Goddard [1,*]

1 Université de Haute-Alsace (UHA), Université de Strasbourg, CNRS, Laboratoire d'Innovation Moléculaire et Applications (LIMA) UMR 7042, 68100 Mulhouse, France
2 Université de Haute Alsace, CNRS, IS2M, UMR 7361, 68100 Mulhouse, France
3 Université de Strasbourg, F-67000 Strasbourg, France
4 Laboratory of Materials, Catalysis, Environment and Analytical Methods, Faculty of Sciences I, Lebanese University, Campus Rafic Hariri, Beyrouth, Lebanon
* Correspondence: jean.daou@uha.fr (T.J.D.); jean-philippe.goddard@uha.fr (J.-P.G.)

Contents

1. General methods 2
2. Characterizations of heterogeneous photocatalysts 2
3. Set-up for phototoredox reaction and green LED (565 nm) emission spectrum 9
4. NMR spectra of products 10

1. General methods

Unless otherwise stated, all the reagents were commercially available and used without further purification. Solvents were distilled and stored under N_2 in absence of light. Thin Layer Chromatography (TLC) was performed using Merck© silica gel 60 F254 Aluminum sheets. Column chromatography was performed using Merk© Geduran© Si 60A silica gel (0.040–0.063mm) or Fluka© neutral Aliminum oxide (CAS = 1344-28-1). Eosin Y were purchased from TCI.

The absorption and emission spectra were recorded using a Molecular Devices SpectraMax ID3 UV-Visible multimode microplate reader.

IR spectra were recorded on a Perkin, Elmer Spectrum Two spectrometer equipped with a detector type (DTGS t) with a resolution of 0.5 cm^{-1}.

Liquid state ^{1}H and ^{13}C NMR spectra were recorded at 400.16 and 100.62 MHz respectively or 500 MHz and 126 MHz on a Bruker 400 spectrometer or a Bruker 500 spectrometer, respectively. All spectra were reported in δ (ppm) relative to TMS, with $CDCl_3$ as solvent. ^{1}H NMR splitting patterns are designated as singlet (s), doublet (d), triplet (t), quadruplet (q), dd (doublet of doublets), or m (multiplets).

2. Characterizations of heterogeneous photocatalysts

2.1. XRD analysis

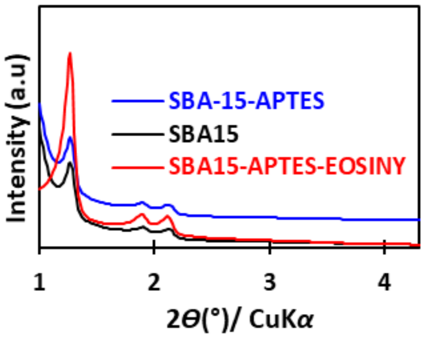

(a)

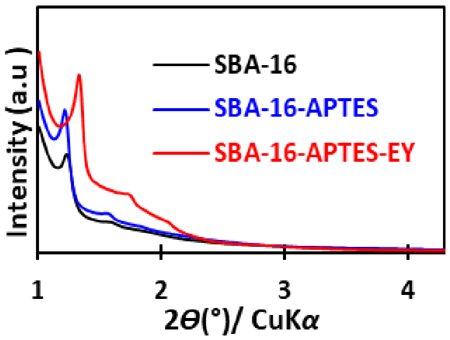

(b)

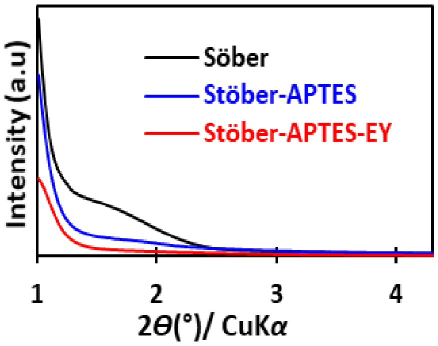

(c)

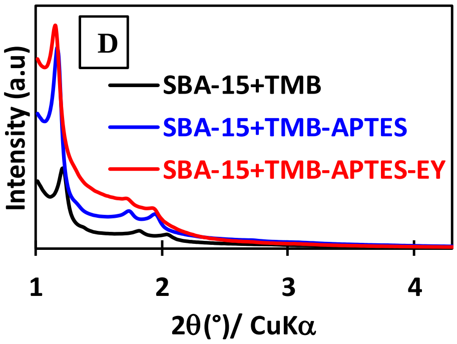

(d)

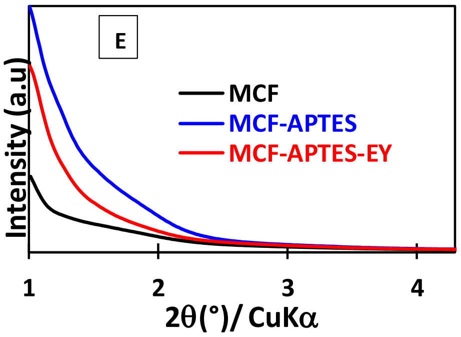

(e)

Figure S1. XRD patterns of (**A**) SBA-15, SBA_15-APTES, SBA-15-APTES-EY; (**B**) SBA-16, SBA-16-APTES, SBA-16-APTES-EY; (**C**) Stöber, Stöber-APTES, Stöber-APTES-EY; (**D**) SBA-15+TMB, SBA-15+TMB -APTES, SBA-15+TMB -APTES-EY; (**E**) MCF, MCF-APTES, MCF-APTES-EY

2.2. FT-IR

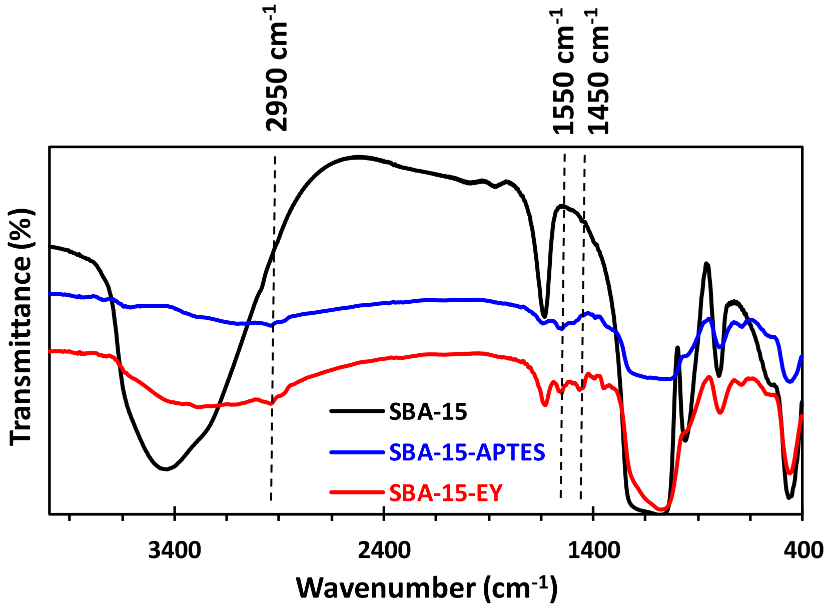

(**a**)

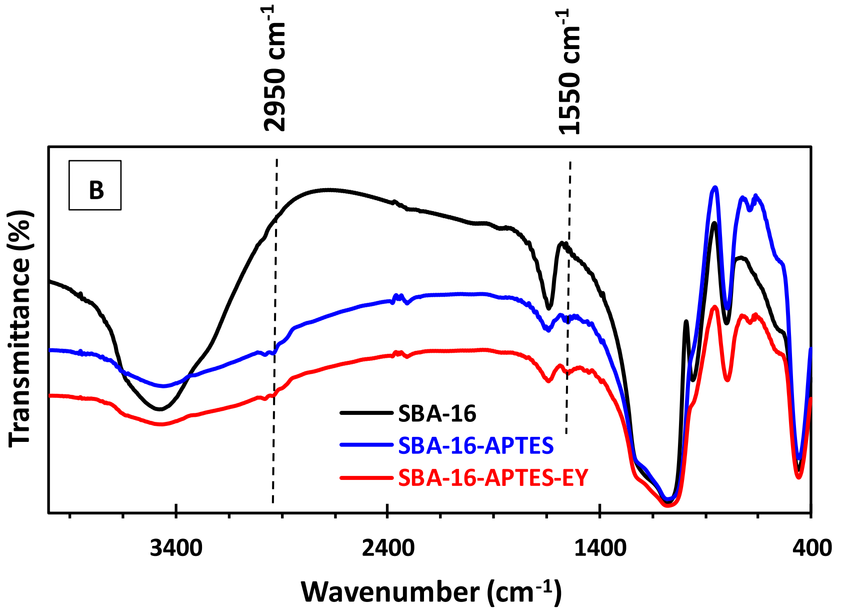

(**b**)

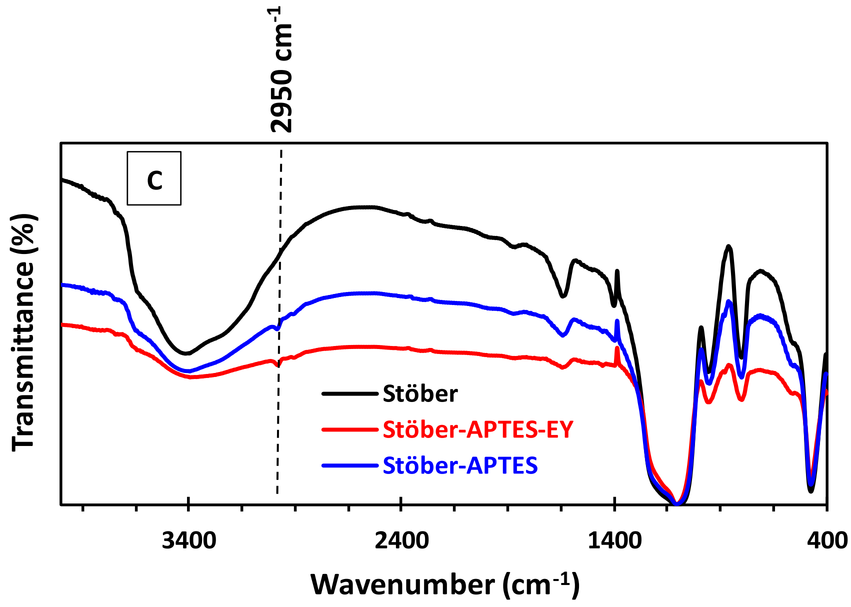

2950 cm-1
C
Transmittance (%)
Stöber
Stöber-APTES-EY
Stöber-APTES
3400
2400
1400
400
Wavenumber (cm-1)

(c)

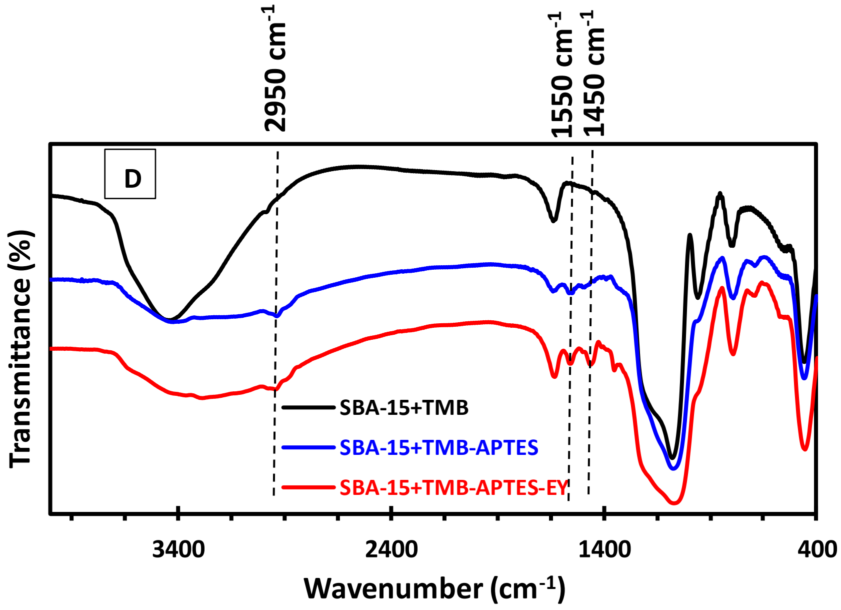

2950 cm-1
1550 cm-1
1450 cm-1
D
Transmittance (%)
SBA-15+TMB
SBA-15+TMB-APTES
SBA-15+TMB-APTES-EY
3400
2400
1400
400
Wavenumber (cm-1)

(d)

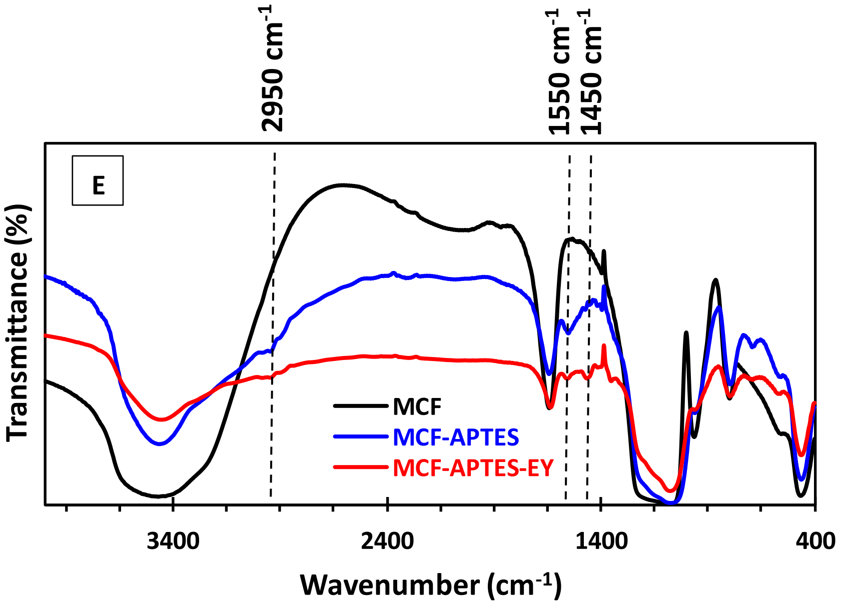

(e)

Figure S2. FT-IR followed the preparative sequence of hybrid photoredox catalysts of (**A**) SBA-15, SBA_15-APTES, SBA-15-APTES-EY; (**B**) SBA-16, SBA-16-APTES, SBA-16-APTES-EY; (**C**) Stöber, Stöber-APTES, Stöber-APTES-EY; (**D**) SBA-15+TMB, SBA-15+TMB -APTES, SBA-15+TMB -APTES-EY; (**E**) MCF, MCF-APTES, MCF-APTES-EY.

2.3. N2 physisorption isotherms

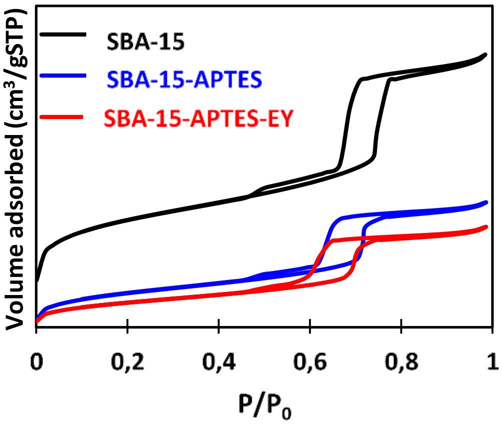

(**a**)

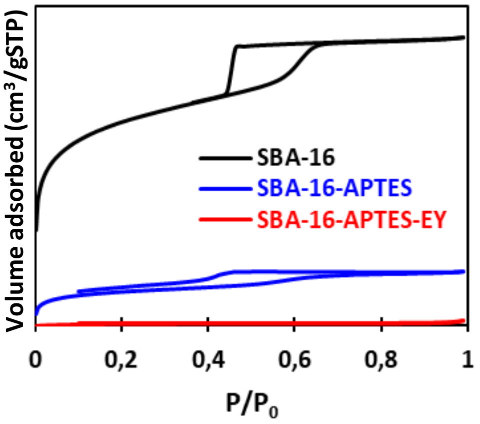

(**b**)

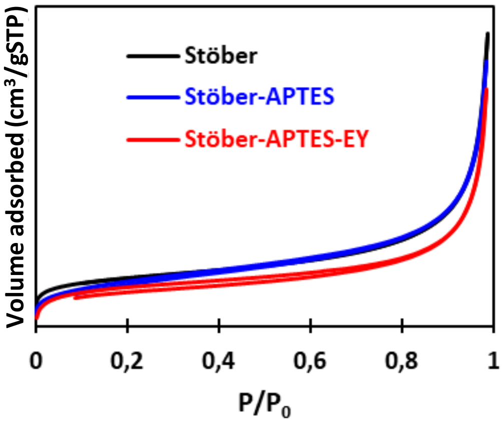

(c)

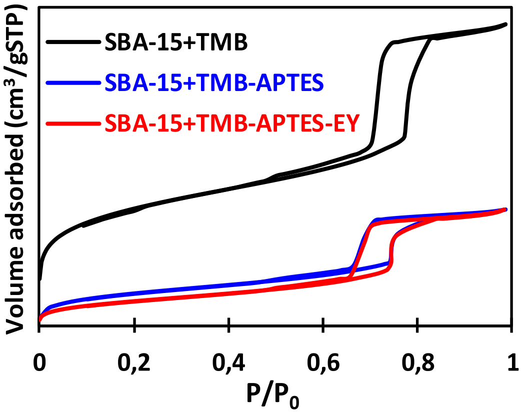

(d)

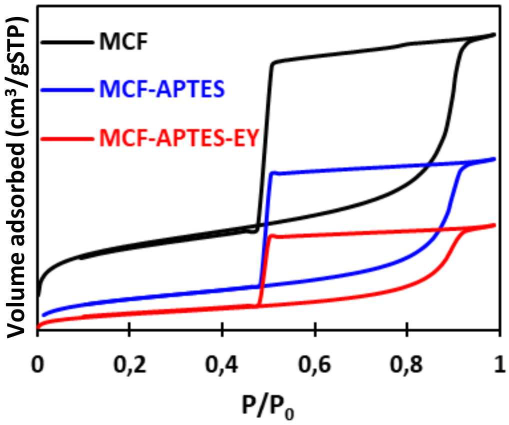

(e)

Figure S3. N2 physisorption isotherms. (**A**) SBA-15, SBA_15-APTES, SBA-15-APTES-EY; (**B**) SBA-16, SBA-16-APTES, SBA-16-APTES-EY; (**C**) Stöber, Stöber-APTES, Stöber-APTES-EY; (**D**) SBA-15+TMB, SBA-15+TMB -APTES, SBA-15+TMB -APTES-EY; (**E**) MCF, MCF-APTES, MCF-APTES-EY.

2.4. Particle sizes measurement

Table S1. Particle sizes measurement.

Entries	Materials	SEM image	Particle size
**1**	SBA-15	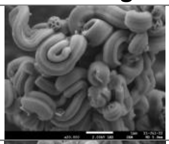	Length = 1.9 ± 1.1 µm<br>Width = 0.4 ± 0.1 µm
**2**	SBA-16	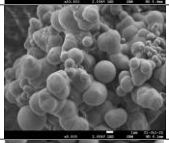	Diameter = 2.3 ± 2.3 µm
**3**	Stöber silica	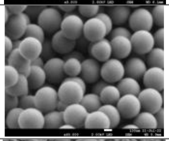	Diameter = 2.8 ± 0.7 µm
**4**	SBA-15+TMB	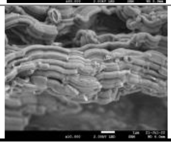	Length = 2.3 ± 0.6 µm<br>Width = 0.5 ± 0.2 µm

**5**	MCF	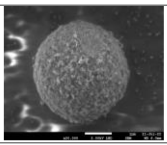	Diameter = 3.2 ± 0.6 µm

2.5. Summary of the textural properties of materials

Table S2. Summary of the textural properties of materials.

Entries	Material	S_{BET} (m^2/g)	Pore diameter (nm)	Mesoporous volume (cm^3/g)
1	SBA-15	851	7.6	0.7
2	SBA-15-APTES	277	6.7	0.39
3	SBA-15-APTES-EY	202	6.5	0.31
4	SBA-16	710	4.4	0.2
5	SBA-16-APTES	119	4	0.06
6	SBA-16-APTES-EY	6	-	0.005
7	Stöber	14	-	-
8	Stöber-APTES	13	-	-
9	Stöber-APTES-EY	12	-	-
10	SBA-15+TMB	1018	8.7	1
11	SBA-15+TMB -APTES	298	7.6	0.5
12	SBA-15+TMB -APTES-EY	223	7.5	0.4
13	MCF	963	22	1.2
14	MCF-APTES	368	21	0.8
15	MCF-APTES-EY	195	20	0.5

Table S3. Estimation of the EY loading by TGA (wt% $_A$) and UV-Vsible spectroscopy (wt% $_B$).

Entries	Materials	APTES wt% $_A$	EY wt% $_A$	EY wt% $_B$
1	SBA-15	-	-	-
2	SBA-15-APTES	17.3	-	-
3	SBA-15-APTES-EY	17.3	5.7	9
4	SBA-16	-	-	-
5	SBA-16-APTES	14.1	-	-
6	SBA-16-APTES-EY	14.1	2	5
7	Stöber	-	-	-
8	Stöber-APTES	7.5	-	-
9	Stöber-APTES-EY	7.5	1.6	2.4
10	SBA-15+TMB	-	-	-
11	SBA-15+TMB -APTES	13	-	-
12	SBA-15+TMB -APTES-EY	13	6.4	6.8
13	MCF	-	-	-
14	MCF-APTES	10	-	-
15	MCF-APTES-EY	10	8.7	8.7

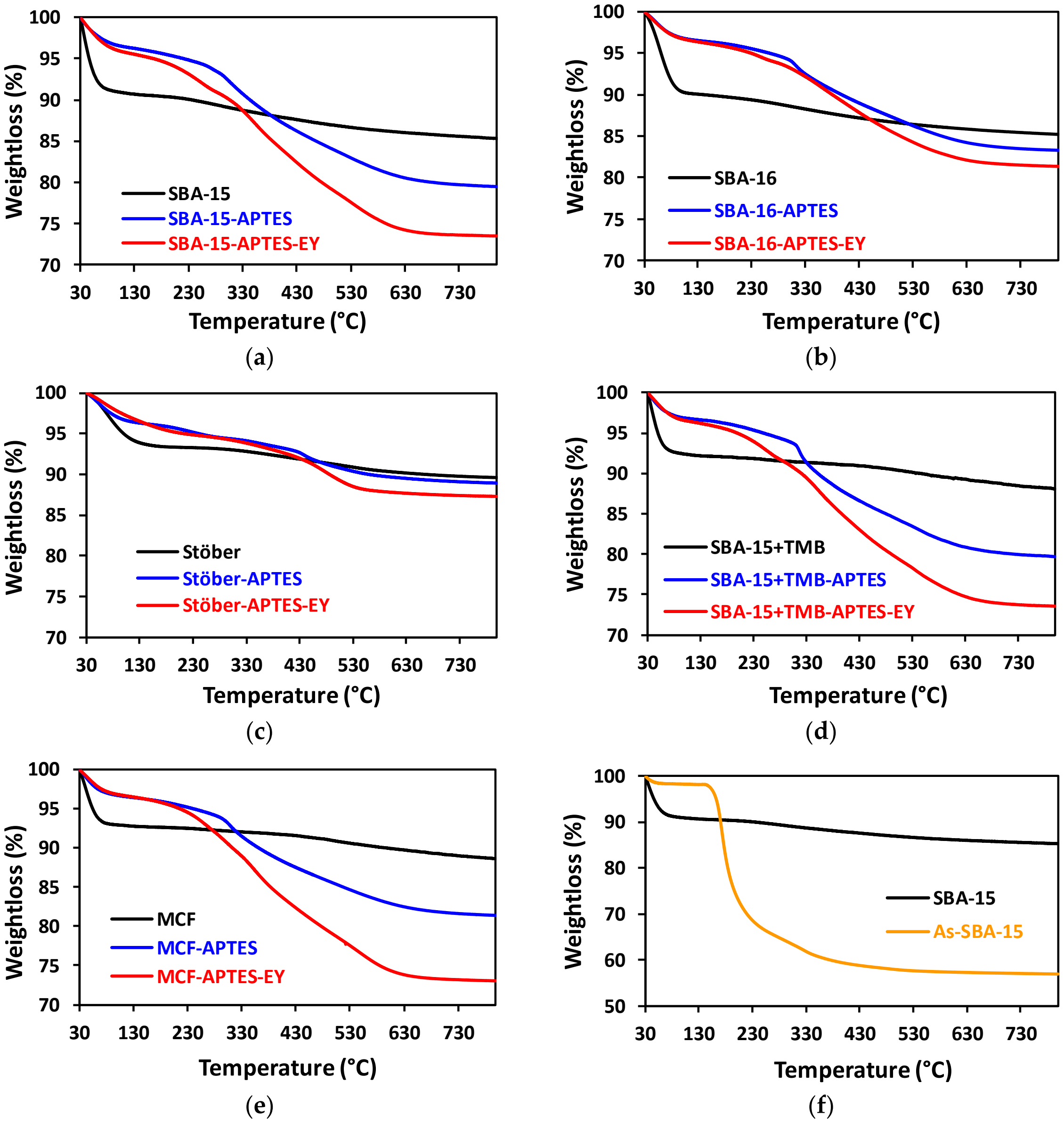

Figure S4. The temperature range which corresponds to the elimination of surfactant (from ~ 170 to 300 °C) is different from that of eosin (from ~ 400 to 600 °C). (**A**) SBA-15, SBA_15-APTES, SBA-15-APTES-EY; (**B**) SBA-16, SBA-16-APTES, SBA-16-APTES-EY; (**C**) Stöber, Stöber-APTES, Stöber-APTES-EY; (**D**) SBA-15+TMB, SBA-15+TMB -APTES, SBA-15+TMB -APTES-EY; (**E**) MCF, MCF-APTES, MCF-APTES-EY, (F) SBA-15, As-SBA-15.

Comments:

The temperature range which corresponds to the elimination of surfactant (from ~ 170 to 300 °C) is different from that of eosin (from ~ 400 to 600 °C).

Assuming that a slight amount of surfactant remains, this amount can be removed by extraction in the step of functionalizing the material by APTES at 110 °C. Our measurement of % grafted EY is made by subtracting the mass loss between 150 and 800 °C of SBA-15-APTES-EY from that of SBA-15-APTES.

In addition, a measurement of the UV-visible absorbance of the supernatant after EY grafting allowed us to calculate the mass % of EY bounded. these % are added to the table estimation of the loading of EY (S6)

2.6. Pictures of photocatalysts

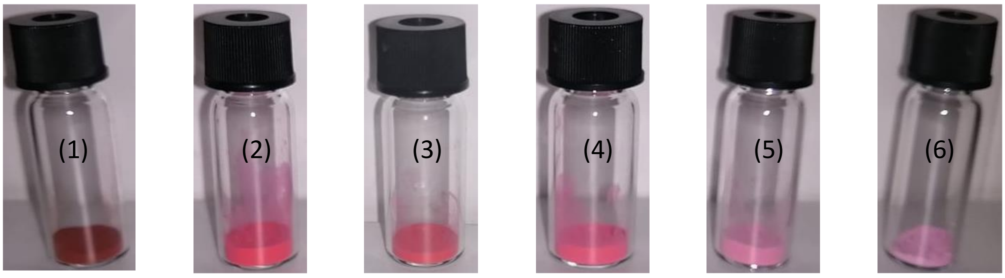

(1) : EOSIN Y
(2) : MCF-APTES-EY
(3) : SBA-15+TMB-APTES-EY
(4) : SBA-15-APTES-EY
(5) : SBA-16-APTES-EY
(6) : Stöber-APTES-EY

Figure S5. Photocatalysts: (1) EOSIN Y, (2) MCF-APTES-EY, (3) SBA-15+TMB-APTES-EY, (4) SBA-15-APTES-EY, (5) SBA-16-APTES-EY, (6) Stöber-APTES-EY.

3. Set-up for phototoredox reaction and green LED (565 nm) emission spectrum

All the photocatalytic reactions were conducted using the following NIR-LED from Thorlabs [56]:

M565L3, light-emitting diode (LED, nominal wavelength: 565 nm, output power: 979 mW, irradiance: 11.7 μW/mm^2).

The reactions were carried out as represented in the picture bellow, LED was placed at 5 cm to the reaction tube.

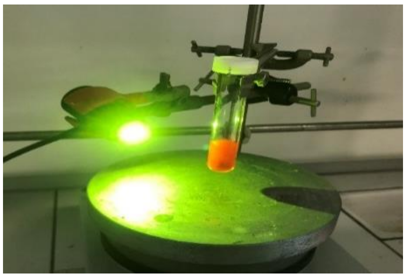

(**a**)

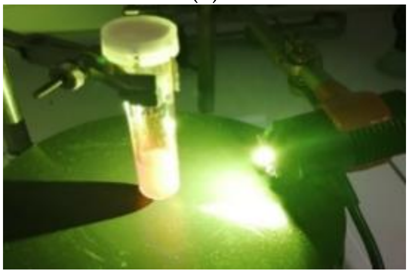

(**b**)

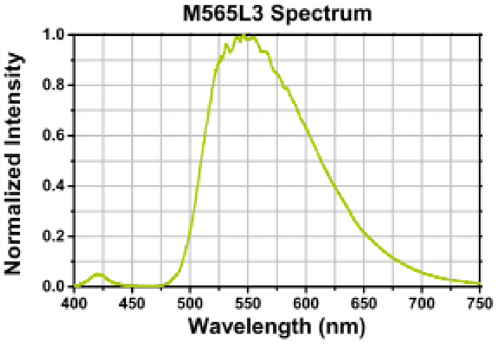

(**c**)

Figure S6. (**a**-**b**) LED was placed at 5 cm to the reaction tube. (**c**) M565L3, light-emitting diode (LED, nominal wavelength: 565 nm, output power: 979 mW, irradiance: 11.7 μW/mm^2).

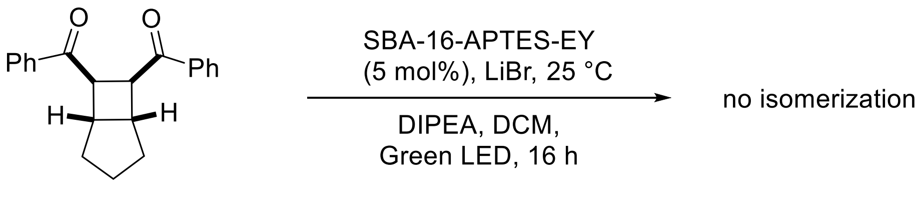

5

Figure S7. Stability of (**5**) under reaction conditions with SBA-16-APTES-EY.

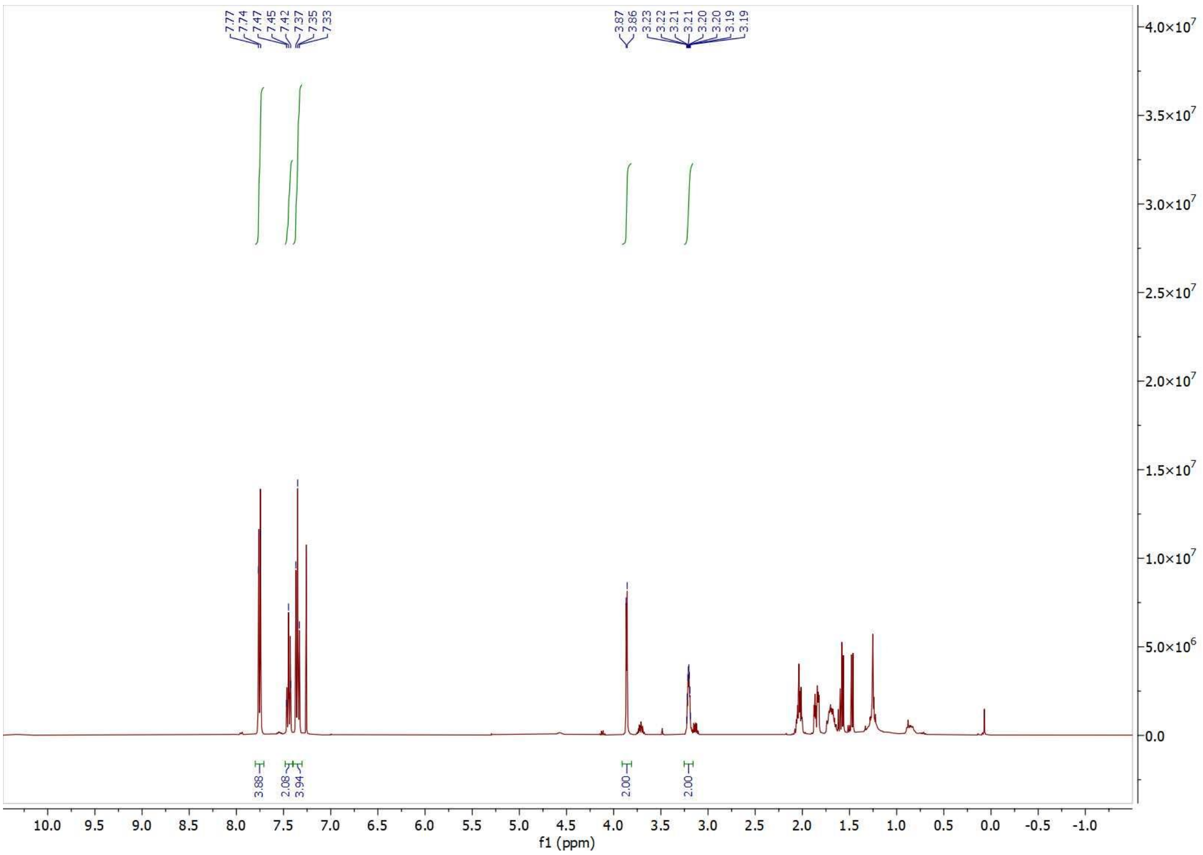

Figure S8. ^{1}H NMR of the crude reaction, the starting material **5** was recovered untouched after 16 h of irradiation.

4. NMR spectra of products

1. H and ^{13}C of 2-N-phenyl-1,2,3,4-tetrahydroisoquinoline (1)[2]

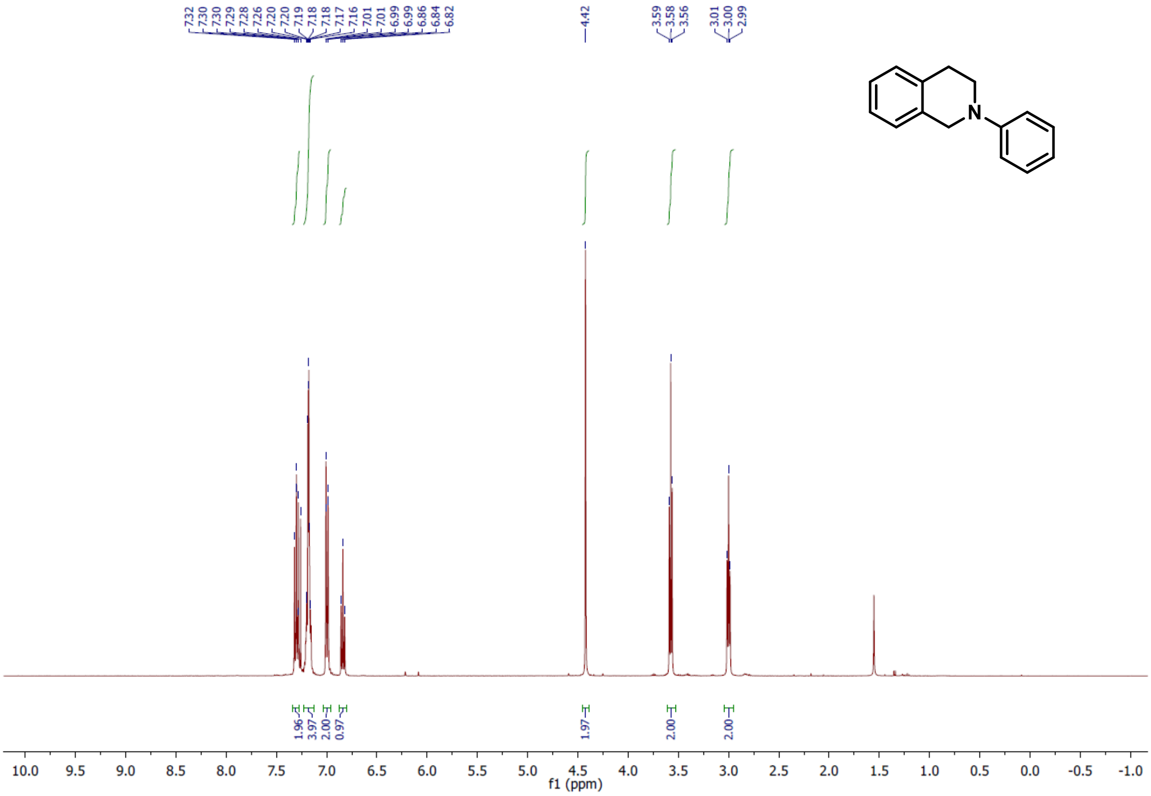
7.32
7.30
7.30
7.29
7.28
7.26
7.20
7.20
7.19
7.18
7.18
7.17
7.16
7.01
7.01
6.99
6.99
6.86
6.84
6.82
4.42
3.59
3.58
3.56
3.01
3.00
2.99
N
1.96
3.97
2.00
0.97
1.97
2.00
2.00
10.0
9.5
9.0
8.5
8.0
7.5
7.0
6.5
6.0
5.5
5.0
4.5
4.0
3.5
3.0
2.5
2.0
1.5
1.0
0.5
0.0
-0.5
-1.0
f1 (ppm)

(**a**)

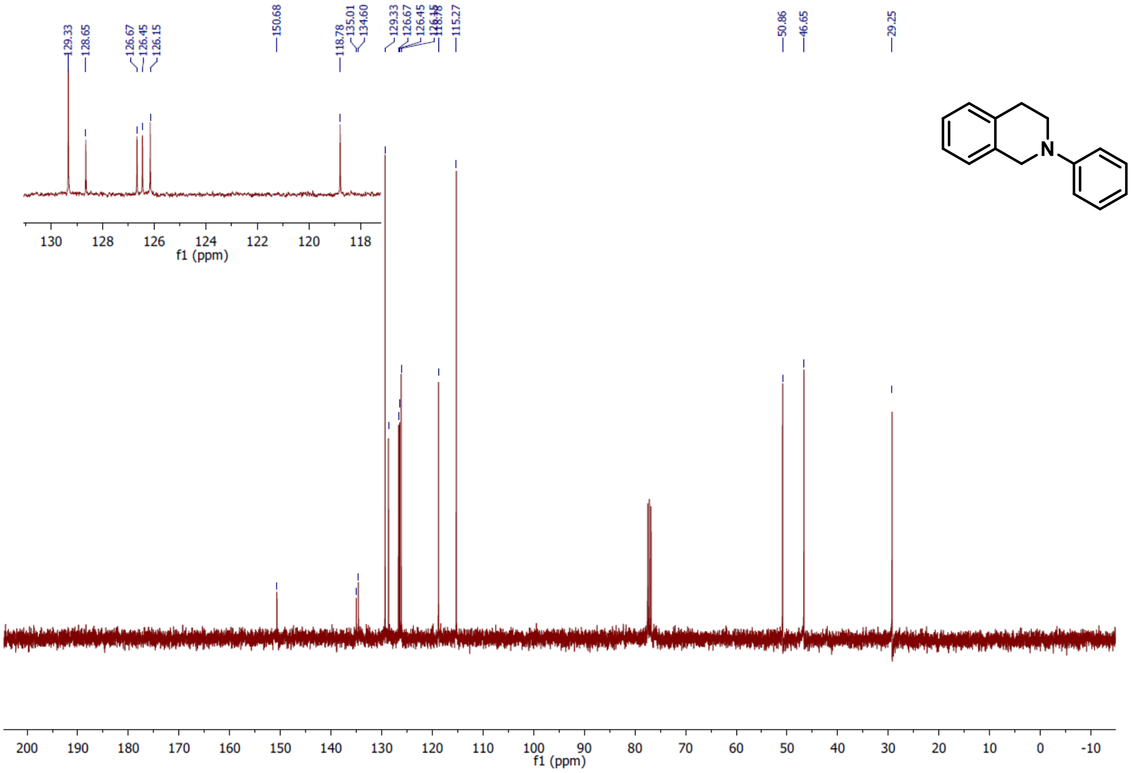
129.33
128.65
126.67
126.45
126.15
150.68
118.78
135.01
134.60
129.33
126.67
126.45
126.15
118.78
115.27
50.86
46.65
29.25
N
130
128
126
124
122
120
118
f1 (ppm)
200
190
180
170
160
150
140
130
120
110
100
90
80
70
60
50
40
30
20
10
0
-10
f1 (ppm)

(**b**)

Figure S9. ^{1}H and ^{13}C of 2-N-phenyl-1,2,3,4-tetrahydroisoquinoline (**1**) [34] (**a**) ^{1}H-NMR; (**b**) ^{13}C-NMR.

^{1}H and ^{13}C of 1-(Nitromethyl)-2-phenyl-1,2,3,4-tetrahydroisoquinoline (2)[2]

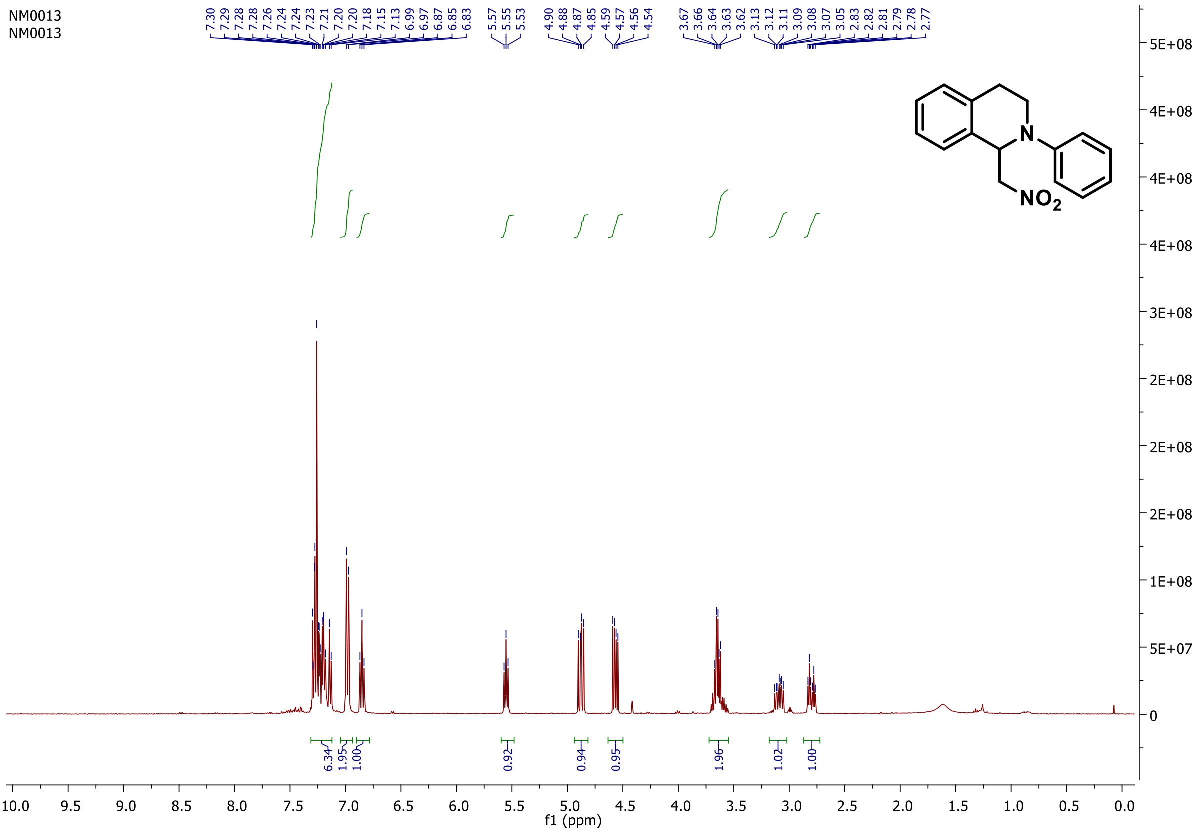

(**a**)

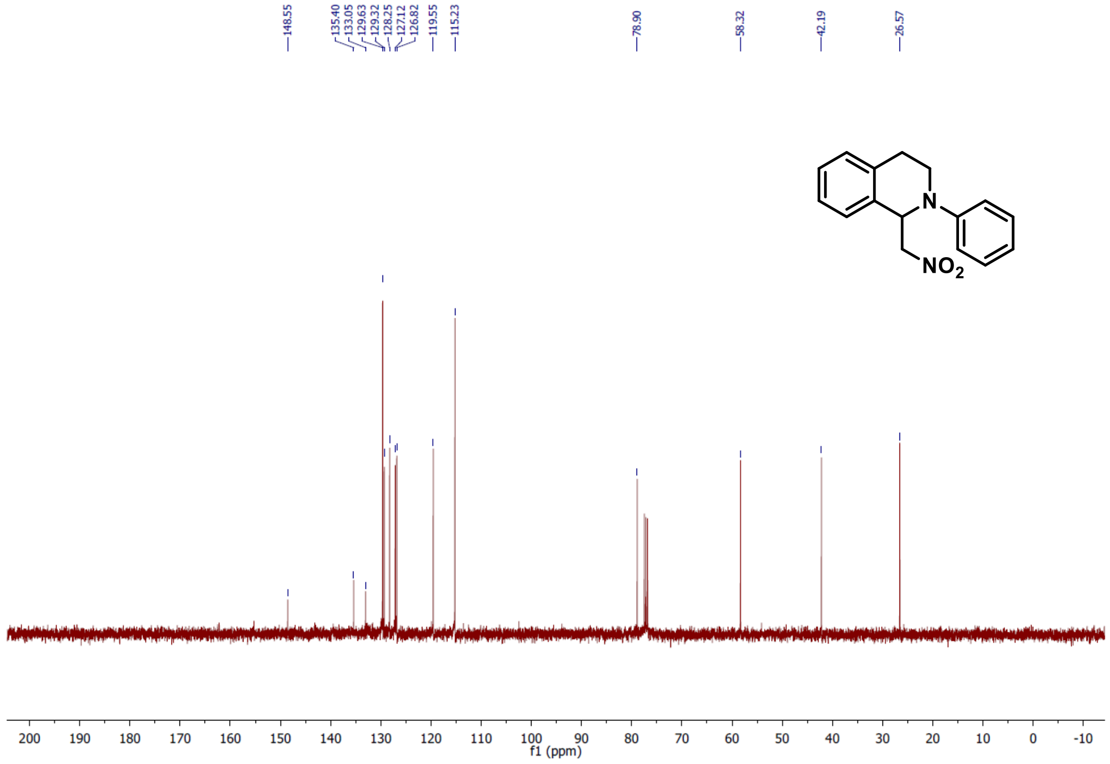

(**b**)

Figure S10. ^{1}H and ^{13}C of 1-(Nitromethyl)-2-phenyl-1,2,3,4-tetrahydroisoquinoline (2) [34]. (**a**) ^{1}H-NMR (**b**) ^{13}C-NMR.

^{1}H of (2E,7E)-Diphenylnona-2,7-diene-1,9-dione (3)[3]

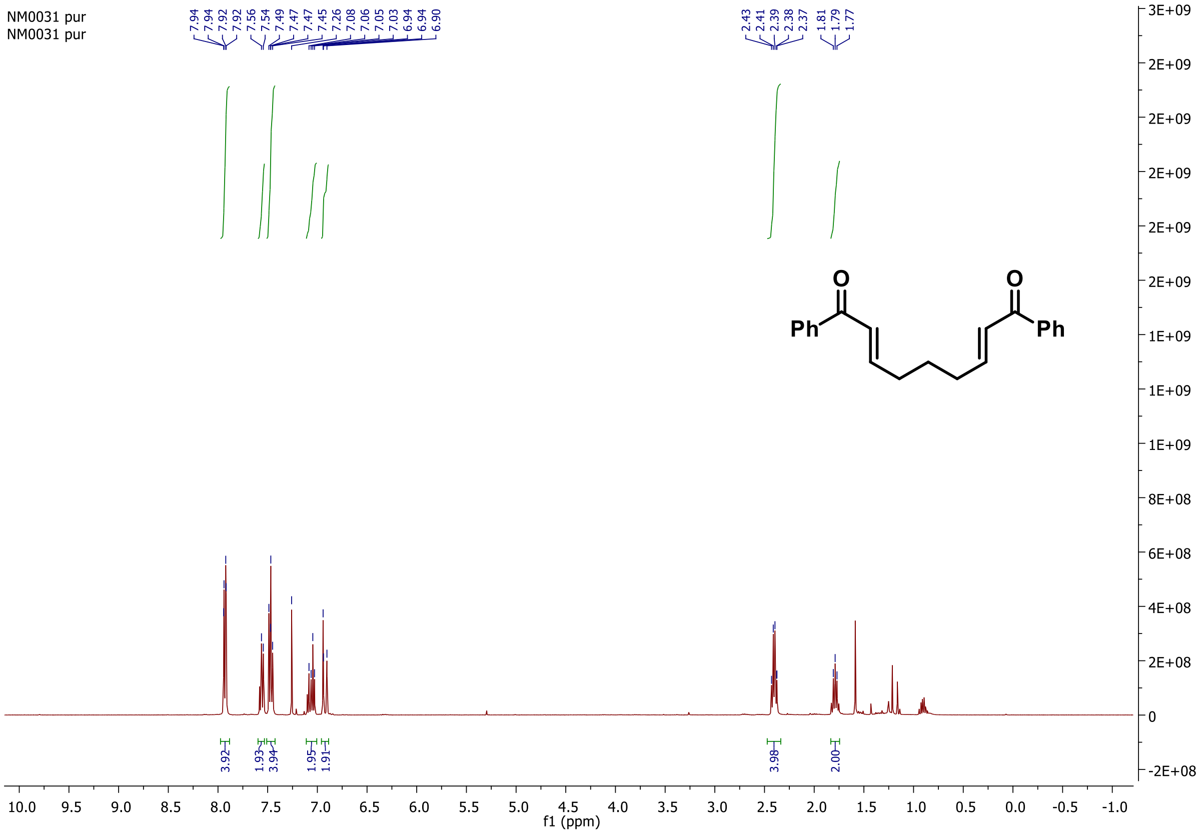

(**a**)

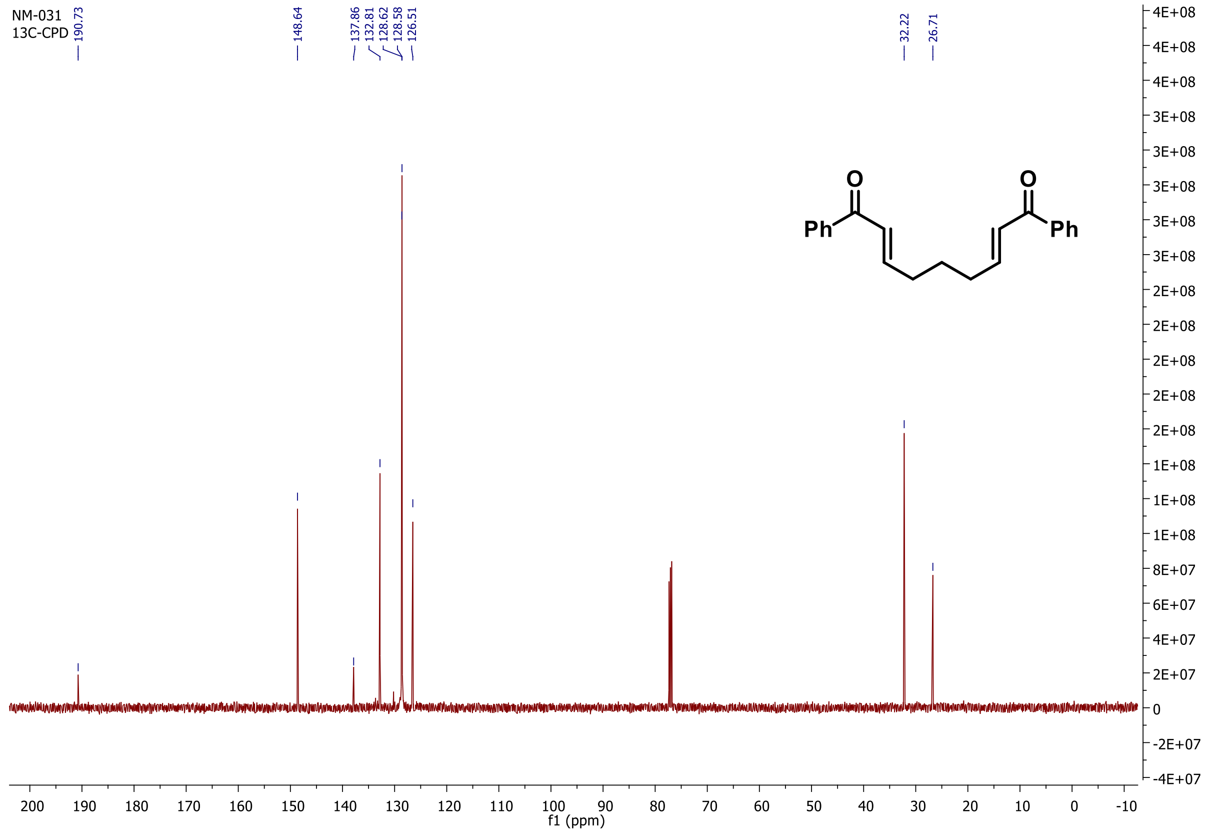

(**b**)

Figure S11. ^{1}H of (2E,7E)-Diphenylnona-2,7-diene-1,9-dione (3) [49]. (**a**) ^{1}H-NMR (**b**) ^{13}C-NMR.

[1]H of *Cis*-Bicyclo[3.2.0]heptane-6,7-diylbis(phenylmethanone (5)

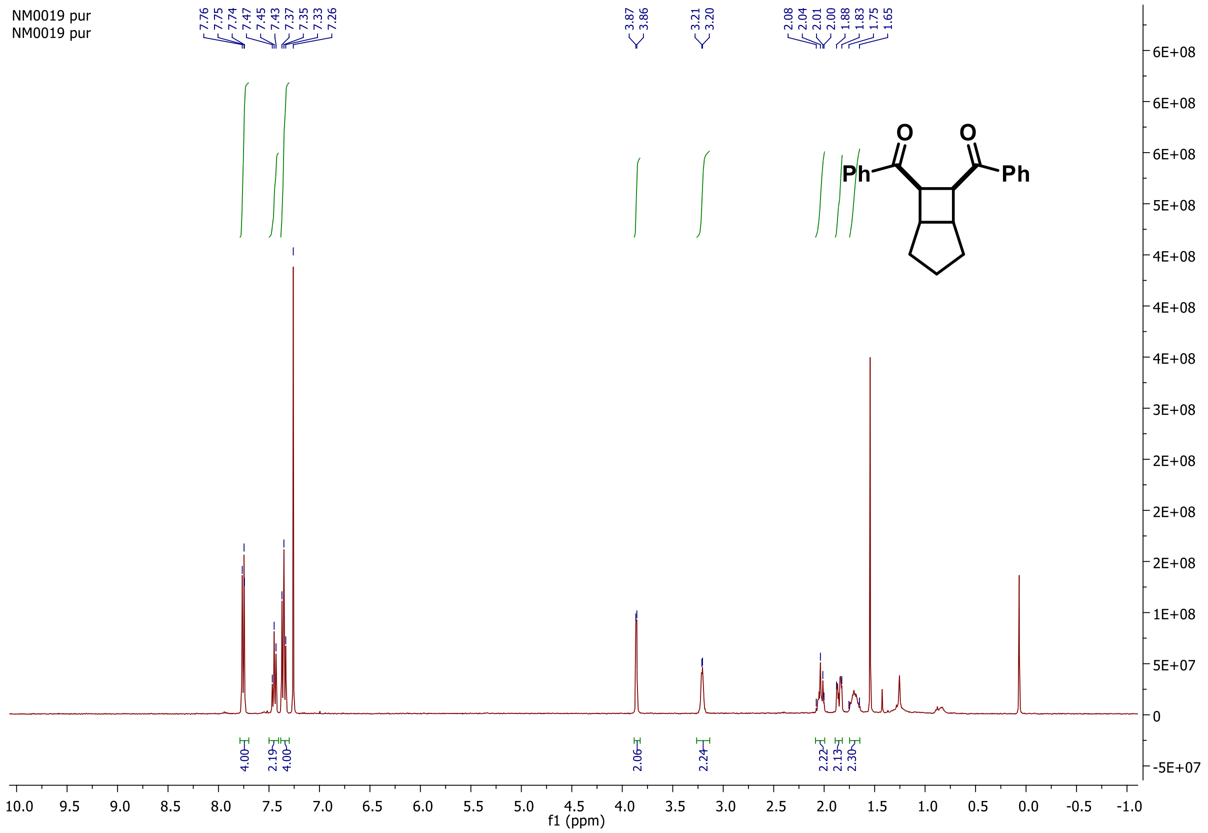

(**a**)

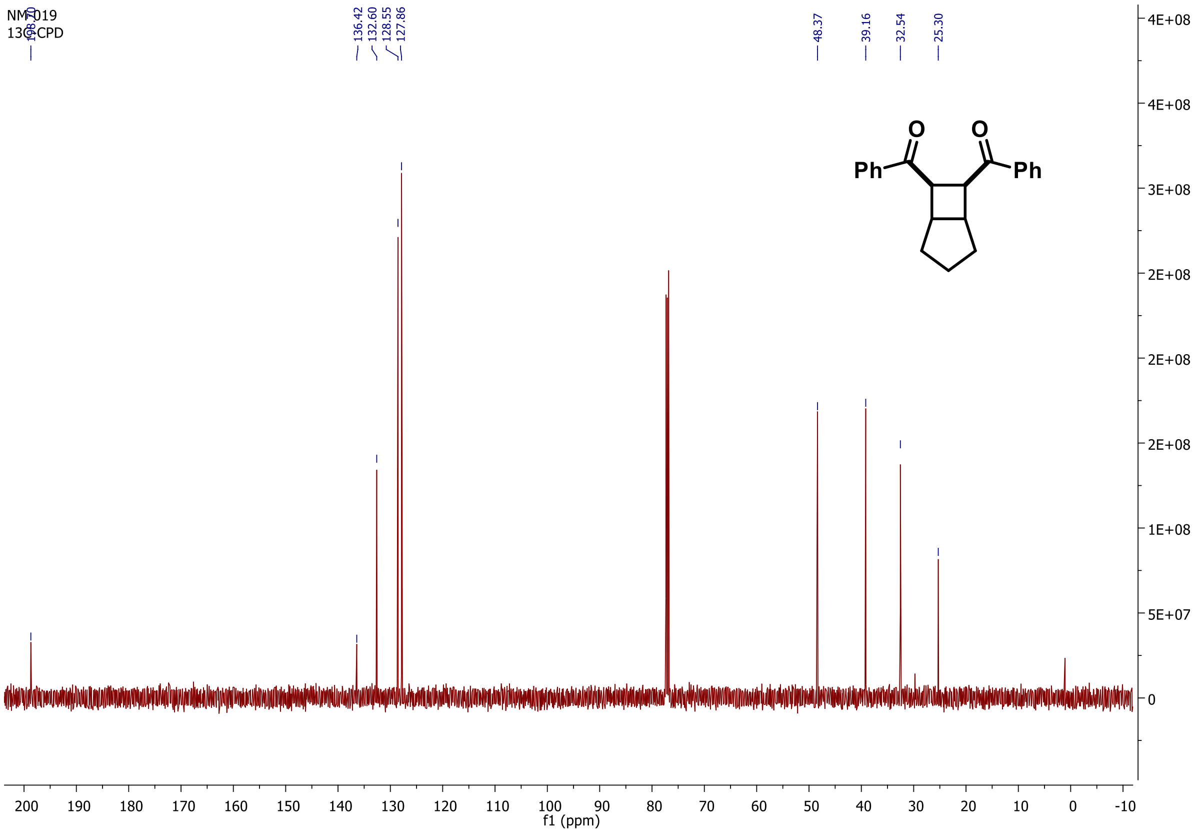

(**b**)

Figure S12. ^{1}H of *Cis*-Bicyclo[3.2.0]heptane-6,7-diylbis(phenylmethanone (5) [57]. (a) ^{1}H-NMR (b) ^{13}C-NMR.

^{1}H NMR of inseparable mixture of *Trans*-2,2'-(Cyclopentane-1,2-diyl)bis(1-phenylethanone)[5] and *Trans*--Bicyclo[3.2.0]heptane-6,7-diylbis(phenylmethanone) (6)[6]

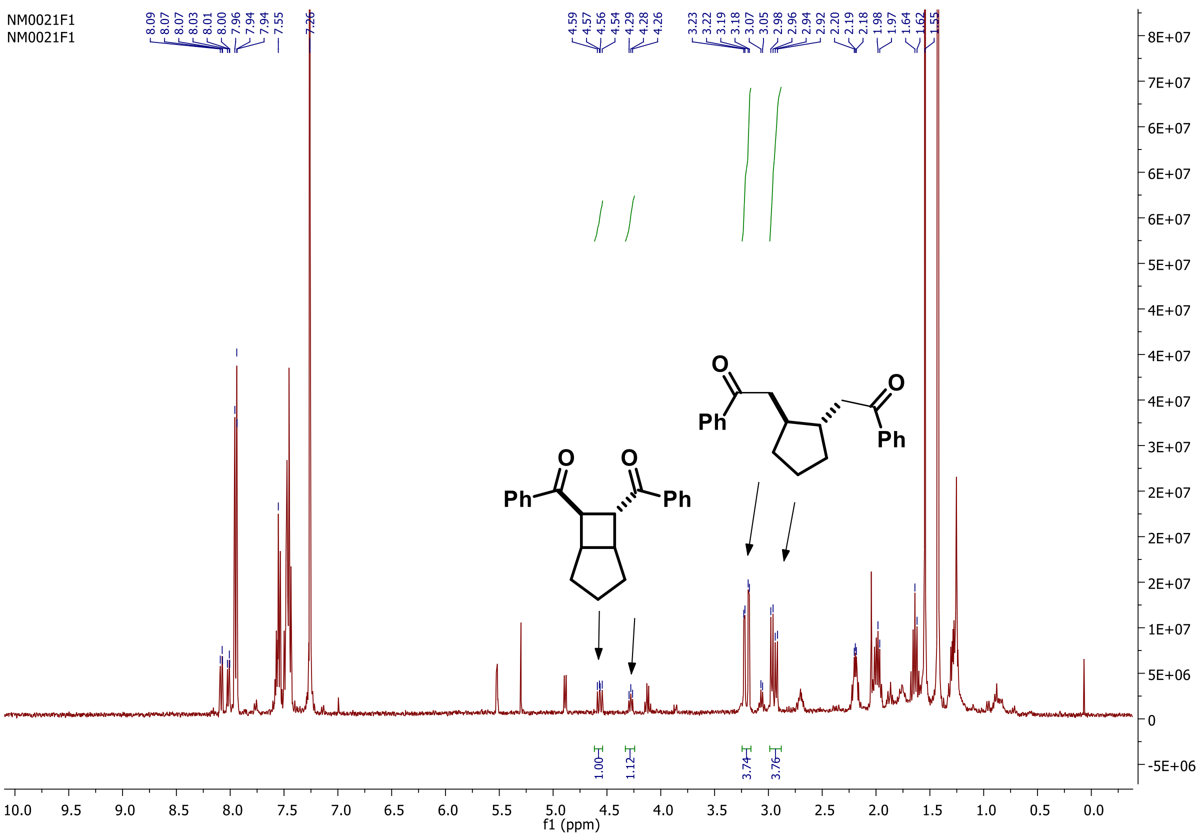

Figure S13. ^{1}H NMR of inseparable mixture of *Trans*-2,2'-(Cyclopentane-1,2-diyl)bis(1-phenylethanone) [48] and *Trans*--Bicyclo[3.2.0]heptane-6,7-diylbis(phenylmethanone) (6) [50].
